# Symmetrically dimethylated histone H3R2 promotes global transcription during minor zygotic genome activation in mouse pronuclei

**DOI:** 10.1038/s41598-021-89334-w

**Published:** 2021-05-12

**Authors:** Kohtaro Morita, Yuki Hatanaka, Shunya Ihashi, Masahide Asano, Kei Miyamoto, Kazuya Matsumoto

**Affiliations:** 1 Laboratory of Molecular Developmental Biology, Graduate School of Biology-Oriented Science and Technology, Kindai University, Wakayama, Japan; 2 Institute of Laboratory Animals, Graduate School of Medicine, Kyoto University, Kyoto, Japan; 3 RIKEN BioResource Research Center, Tsukuba, Ibaraki Japan; 4 Medical Research Council (MRC) London Institute of Clinical Sciences, Imperial College London, London, UK

**Keywords:** Developmental biology, Reprogramming

## Abstract

Paternal genome reprogramming, such as protamine–histone exchange and global DNA demethylation, is crucial for the development of fertilised embryos. Previously, our study showed that one of histone arginine methylation, asymmetrically dimethylated histone H3R17 (H3R17me2a), is necessary for epigenetic reprogramming in the mouse paternal genome. However, roles of histone arginine methylation in reprogramming after fertilisation are still poorly understood. Here, we report that H3R2me2s promotes global transcription at the 1-cell stage, referred to as minor zygotic genome activation (ZGA). The inhibition of H3R2me2s by expressing a histone H3.3 mutant H3.3R2A prevented embryonic development from the 2-cell to 4-cell stages and significantly reduced global RNA synthesis and RNA polymerase II (Pol II) activity. Consistent with this result, the expression levels of *MuERV-L* as minor ZGA transcripts were decreased by forced expression of H3.3R2A. Furthermore, treatment with an inhibitor and co-injection of siRNA to *PRMT5* and *PRMT7* also resulted in the attenuation of transcriptional activities with reduction of H3R2me2s in the pronuclei of zygotes. Interestingly, impairment of H3K4 methylation by expression of H3.3K4M resulted in a decrease of H3R2me2s in male pronuclei. Our findings suggest that H3R2me2s together with H3K4 methylation is involved in global transcription during minor ZGA in mice.

## Introduction

Fertilised oocytes undergo various and essential events for preimplantation development, such as Ca^2+^ oscillations, meiosis resumption, second polar body extrusion, protamine–histone exchange, and epigenetic reprogramming^1,2^. In particular, the male genome undergoes protamine–histone exchange immediately after fertilisation and is actively demethylated by TET3-dependent and -independent mechanisms at the 1-cell stage, whereas the female genome mainly undergoes DNA replication-dependent passive demethylation up to the morula stage^3–7^. Histone variant H3.3 is predominantly incorporated into male genomes by histone chaperone HIRA at an early pronuclear (PN) stage before DNA replication in zygotes. A deficiency of HIRA prevents the male PN formation and results in incomplete nucleosome assembly^8^, indicating that the recruitment of H3.3 is essential for further events such as reconstitution of paternal genome followed by global transcription. Thereafter, zygotic transcripts are globally increased in a process referred to as zygotic genome activation (ZGA). A large number of protein-coding genes are transcribed at major ZGA during the 2-cell stage, whereas transcription is already initiated at minor ZGA during the late 1-cell stage^7,9–11^. In this reprogramming process, some histone modifications occur during major initial events for embryonic development and genomic stability. A previous report showed that methylation of paternal histone H3K4 was required for minor ZGA^12^. In addition, paternal pericentric regions showed the absence of H3K9me3 and the accumulation of H3K27me3 and H3K9me1, whereas H3K9me2/3 was enriched at maternal pericentric regions^13,14^. HP1-beta is associated with H3K9me1, and thus maintains genomic stability in early embryos^15^. These reports indicate that histone modifications play an invaluable role in paternal chromatin remodelling for global transcription and genome stability at late PN stages.

Recently, we have shown that Mettl23-catalysed asymmetric dimethylation of histone H3R17 (H3R17me2a) is a key regulator for paternal genome reprogramming such as protamine–histone exchange and DNA demethylation in mouse zygotes^16^. The protein arginine methyltransferase (PRMT) family comprises type I (PRMT1, 2, 3, 4, 6, 8) and type II (PRMT5, 7, 9), which catalyse asymmetric and symmetric dimethylation of histone arginine residues, respectively^17–19^. A recent proteomic analysis showed that PRMT7 in the reprogramming candidates is the most abundant protein in preimplantation embryos^20^. PRMT5, which has a similar function, is also abundantly expressed^20^. It is known that PRMT5 catalyses symmetric dimethylation of histones H2AR3, H3R2, H3R8, and H4R3 (H2AR3me2s, H3R2me2s, H3R8me2s, and H4R3me2s), and PRMT7 also catalyses H3R2me2s, H3R8me2s, and H4R3me2s^17,21^. However, the role of these histone arginine methylations, which are catalysed by PRMT5 and PRMT7 in the zygote, is still not fully understood.

Here, we have showed that H3R2me2s is involved in global transcription by mediating the activation of RNA polymerase II (Pol II) in male pronuclei using dominant negative mutants of H3, a dual inhibitor of PRMT5-PRMT7, and siRNAs targeted to these genes. Interestingly, forced expression of H3.3K4M led to significantly reduce the level of H3R2me2s, while H3K4me3 was not altered in H3.3R2A-expressed zygotes. These results suggest that H3R2me2s regulates global transcription during minor ZGA in a H3K4 methylation-dependent manner in zygotes.

## Results

### The accumulation of H3.3R2A in male pronuclei leads to developmental arrest at the 2-cell stage

**Figure 1:**
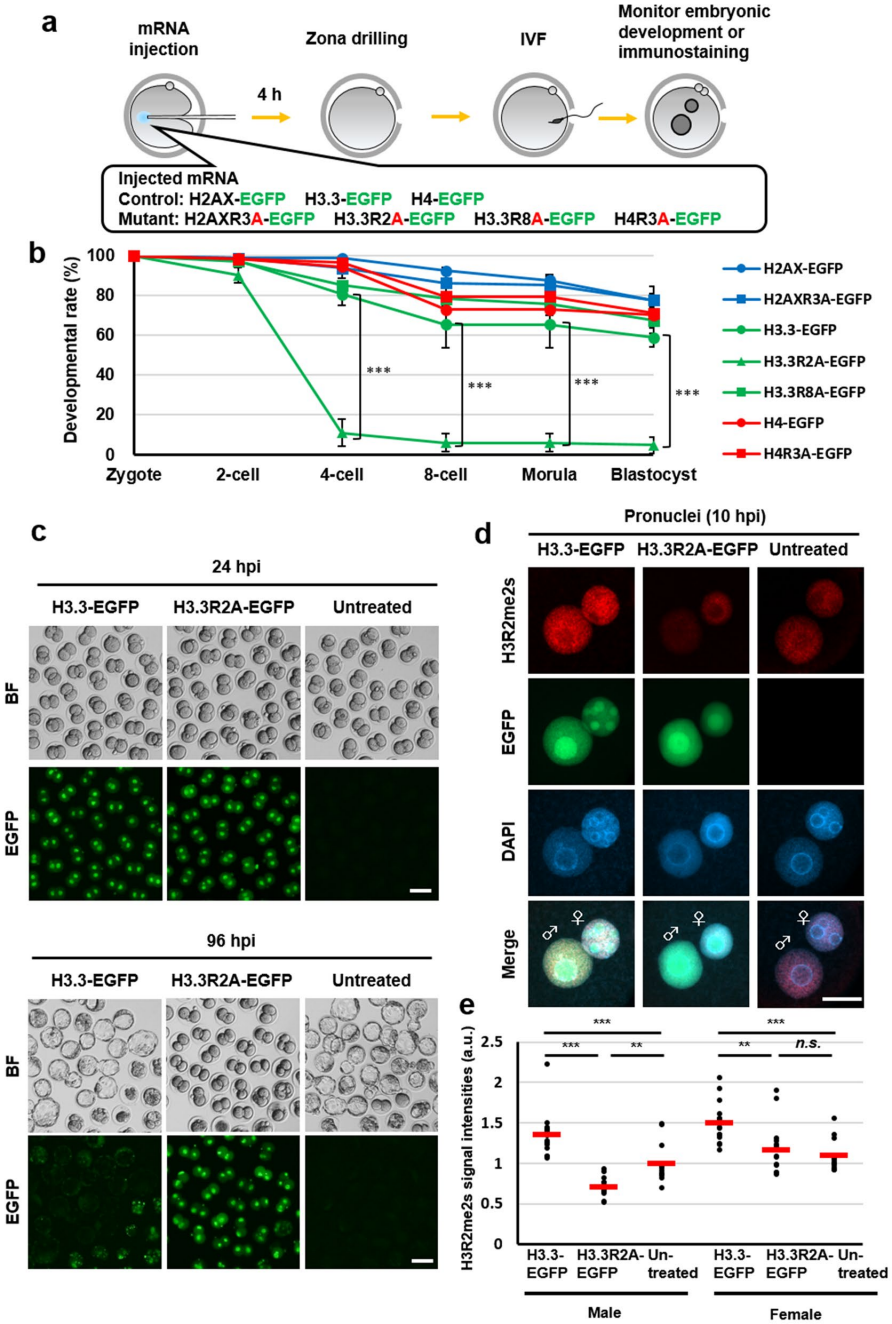
Identification of histone arginine residues for early embryonic development. (**a**) Schematic diagram of the experimental procedures. (**b**) Development of H2AX-EGFP—(blue bar with round box), H2AXR3A-EGFP—(blue bar with square box), H3.3-EGFP—(green bar with round box), H3.3R2A-EGFP—(green bar with triangular box), H3.3R8A-EGFP—(green bar with square box), H4-EGFP—(red bar with round box), and H4R3A-EGFP—(red bar with square box) expressed embryos. A chi-squared test was performed for analysing embryonic development through to the blastocyst stage. The asterisk indicates statistical significance (***p < 0.001). (**c**) Representative images of H3.3-EGFP-expressed, H3.3R2A-EGFP-expressed, and untreated embryos at 24 hpi and 96 hpi. Scale bar = 100 µm. (**d**) Immunofluorescent images of H3R2me2s (red) and DAPI (blue) in H3.3-EGFP-expressed, H3.3R2A-EGFP-expressed, and untreated zygotes. EGFP (green) shows successful injection. Key: ♂, male pronuclei; ♀, female pronuclei. Scale bar = 20 µm. (**e**) Quantification of H3R2me2s signal intensities in the pronuclei of H3.3-EGFP-expressed, H3.3R2A-EGFP-expressed, and untreated zygotes. Each dot plot represents a single zygote. Red bars indicate the mean values. The mean value of male pronuclei in untreated zygotes was set as 1. One-way ANOVA and the Tukey–Kramer test were performed to evaluate statistical significance. *Significantly different from the control (**p < 0.01, ***p < 0.001); *n.s.*: Not significantly different from the control. The number of zygotes was 15 for the H3.3-EGFP group, 15 for the H3.3R2A-EGFP group, and 13 for the untreated group. The pronucleus used for quantification are presented in Fig. S5.

To clarify the role of histone arginine methylation catalysed by PRMT5 and PRMT7 in preimplantation embryos, we examined the effect of forced expression of unmethylatable mutants of arginine residues of H2AX, H3.3, and H4: H2AXR3A, H3.3R2A, H3.3R8A, H4R3A on mouse embryonic development (Fig. 1a). Because PRMT5 and PRMT7 are abundantly expressed in unfertilised mouse oocytes^20,22^, we hypothesised that the corresponding histone post-transcriptional modifications could be important for early embryonic development prior to implantation. Based on this hypothesis, we injected mRNA for point-mutated, enhanced green fluorescent protein (EGFP)-tagged histones into mouse oocytes before fertilisation (Fig. 1a). First, we evaluated the optimal concentration of mRNA for microinjection as 50 ng/µL for each mRNA (Fig. S1 and Table S1). Moreover, we confirmed that the EGFP signals (H2A.X-EGFP, H3.3-EGFP, H4-EGFP, H2A.XR3A-EGFP, H3.3R2A-EGFP, H3.3R8A-EGFP, and H4R3A-EGFP) were observed in MII oocytes and the pronuclei of zygotes at similar levels (Fig. S2). Next, we observed the effects of these mutations on preimplantation development. The forced expression of H3.3R2A-EGFP resulted in developmental arrest at the 2-cell stage unlike in other mutants (Figs. 1b, c, S3 and Table S2). H3.3-EGFP and H3.3R2A-EGFP were preferentially recruited to the male genome at fertilisation (Fig. S4) and were localised in both male and female pronuclei at 10 hpi, PN4 stage (Figs. 1d, e and S5), consistent with the localisation of forced H3.3 expression in previous reports^12,23,24^. We also observed that the incorporation of these H3.3R2A mutants into male pronuclei resulted in diminished H3R2me2s at late PN stages, whereas a marginal difference was detected in female pronuclei (Figs. 1d, e and S5). These results indicate that the reduction of methylation of H3.3R2 in male pronuclei is associated with embryonic development from the 2-cell stage onwards.

### H3R2me2s is localised in the euchromatic region during zygote development

**Figure 2:**
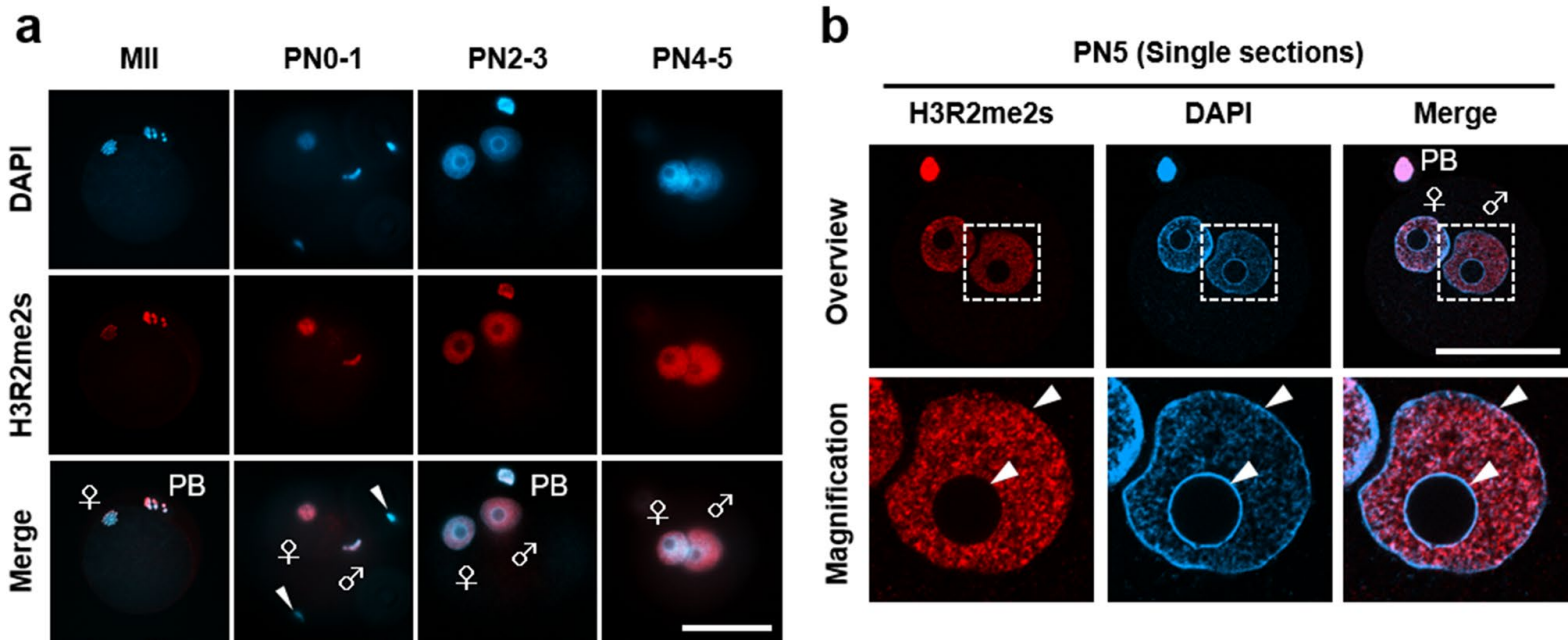
Localisation of H3R2me2s in pronuclei of zygotes. (**a**) Immunostaining for localisation of H3R2me2s in MII oocytes and PN1–5 stage zygotes. The representative images of zygotes stained with DAPI (blue) and anti-H3R2me2s antibody (red). (**b**) An image example showing the male pronucleus of a zygote at PN5. Male pronuclei corresponding to dashed squares are shown magnified below. Shown are representative images of zygotes stained with DAPI (blue) and anti-H3R2me2s antibody (red). Key: ♂, male pronuclei; ♀, female pronuclei; PB, polar body; arrowhead, sperm. Scale bar = 50 µm.

Next, we investigated the localisation profile of H3R2me2s in mouse zygotes. Endogenous H3R2me2s was observed on MII chromosomes and was localised in both pronuclei of zygotes (Fig. 2a). Moreover, the signals were clearly detected in decondensed sperm heads in fertilised oocytes at PN0 but not in sperm heads in the perivitelline space (Fig. 2a). These results indicate that H3R2me2s is incorporated into male pronuclei immediately after fertilisation. Previous reports have shown that H3R2me2s is localised in DAPI-weak euchromatin but not in DAPI-dense heterochromatin, including the chromocenter in *Drosophila* polytene chromosomes^21,25–27^. Consistent with these reports, H3R2me2s signals in mouse zygotes were enriched in DAPI-weak regions but hardly detected in centromere/pericentromere regions and the nuclear periphery (Fig. 2b), which are heterochromatin regions^28–30^. Conversely, asymmetrically dimethylated H3R2 (H3R2me2a), which has been reported as a transcriptional repression marker, was hardly observed in male pronuclei, while it was slightly observed at low levels in a part of the centromere/pericentromere regions (Fig. S6). To summarize, H3R2me2s is localised in euchromatic regions in mouse zygotes in which it may play a role in transcriptional activation rather than H3R2me2a.

### Incorporation of the H3.3R2A mutant into male pronuclei impairs global transcription during minor ZGA

**Figure 3:**
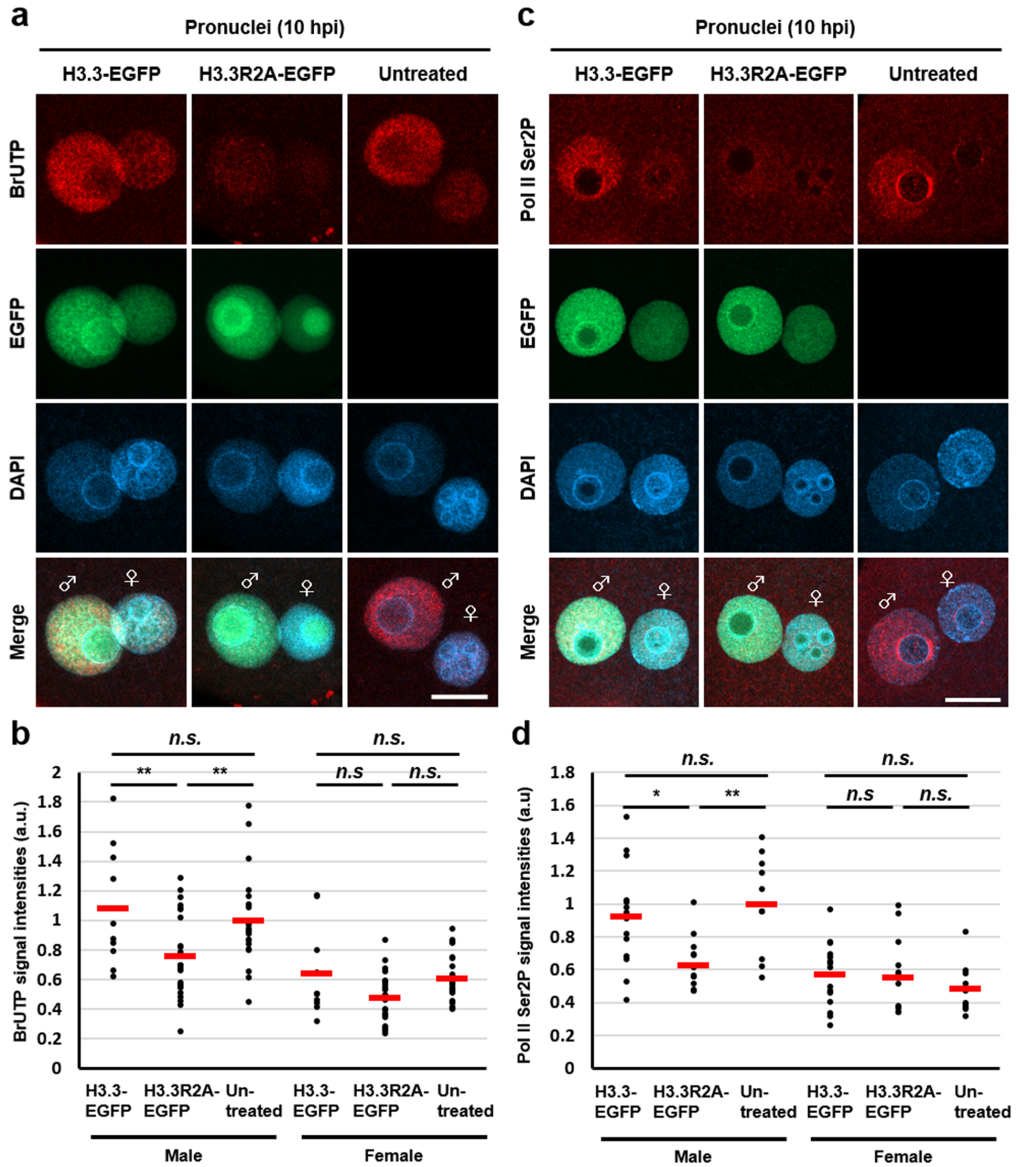
Reduction of transcriptional activities by expression of H3.3R2A mutant in zygotes. (**a**), (**c**) Immunofluorescent images of BrUTP (red), Pol II Ser2P (red), and DAPI (blue) in H3.3-EGFP-expressed, H3.3R2A-EGFP-expressed, and untreated zygotes. EGFP (green) shows successful injection. Key: ♂, male pronuclei; ♀, female pronuclei. Scale bar = 20 µm. (**b**), (**d**) Quantification of the BrUTP and Pol II Ser2P signal intensities in pronuclei of H3.3-EGFP-expressed, H3.3R2A-EGFP-expressed, and untreated zygotes. Red bars indicate the mean values. The mean value of male pronuclei in untreated zygotes was set as 1. One-way ANOVA and the Tukey–Kramer test were performed to evaluate statistical significance. *Significantly different from the control (*p < 0.05, **p < 0.01); *n.s.:* Not significantly different from the control. The number of zygotes in (**b**) was 10 for the H3.3-EGFP expressed group, 24 for the H3.3R2A-EGFP expressed group, and 21 for the untreated group. The number of zygotes in (**d**) was 15 for the H3.3-EGFP expressed group, 13 for the H3.3R2A-EGFP expressed group, and 10 for the untreated group. The pronucleus used for quantification are presented in Fig. S7a, b.

**Figure 4:**
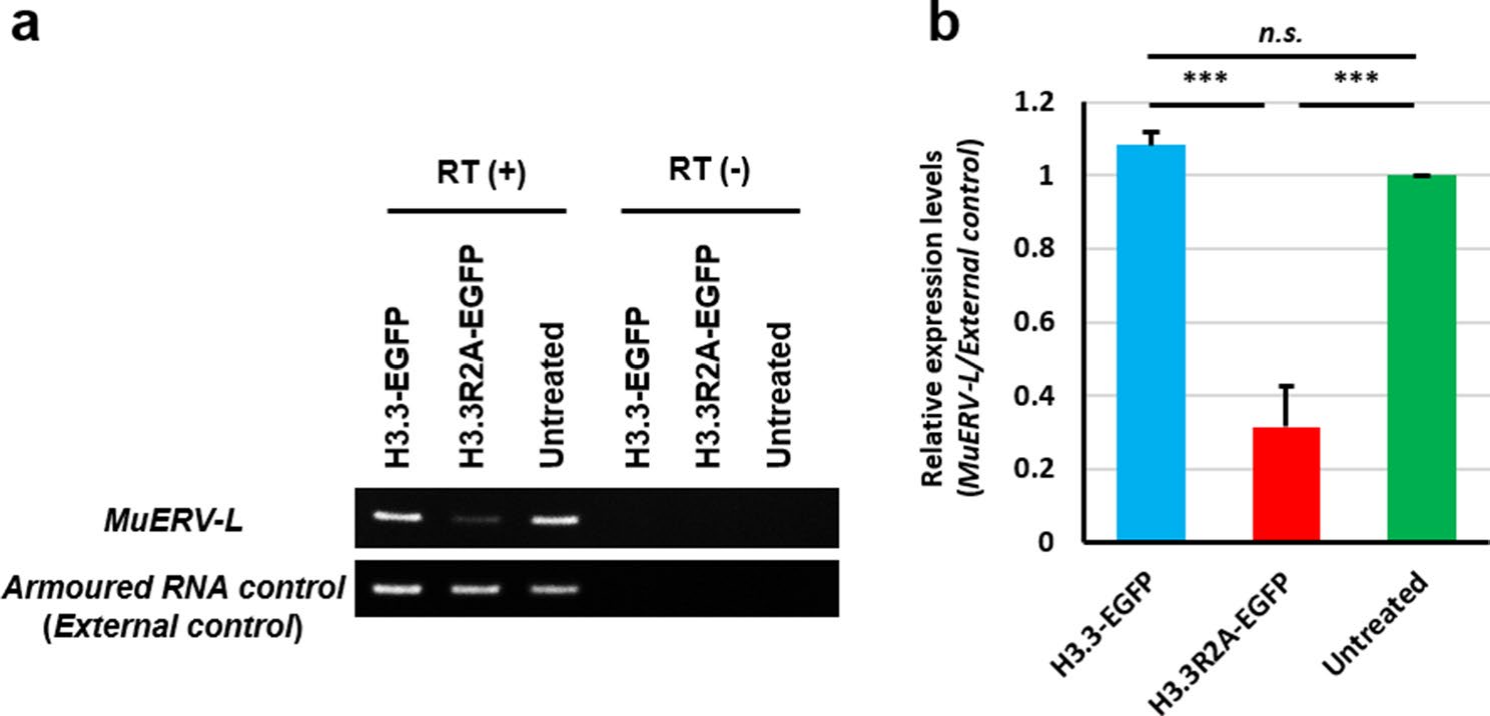
Downregulation of *MuERV-L* via expression of H3.3R2A mutant in early 2-cell at 24 hpi. (**a**) Representative images of RT-PCR of *MuERV-L* and *Armoured RNA control (External control)*. (**b**) Quantification of the PCR bands. The mean value of male pronuclei in untreated zygotes was set as 1. Mean ± S.E. from three independent experiments. One-way ANOVA and the Tukey–Kramer test were performed to evaluate statistical significance. *Significantly different from the control (***p < 0.001); *n.s*.: Not significantly different from the control. The full-length gels used for quantification are presented in Fig. S8.

A recent study showed that the inhibition of minor ZGA by treatment with 5,6-dichloro-1-β-D-ribofuranosylbenzimidazole (DRB), an inhibitor of Pol II elongation, resulted in developmental arrest at the 2-cell stage^31^, implying that a failure in minor ZGA is associated with the 2-cell arrest phenotype. We also observed that embryos that expressed H3.3R2A, in which H3R2me2s was diminished, were arrested at the 2-cell stage (Fig. 1b, c). Therefore, we hypothesised that there might be some relationships between H3R2me2s and minor ZGA. To test this possibility, we examined whether the inhibition of H3R2me2s results in impaired global transcription at minor ZGA. Intranuclear BrUTP signals, which indicate active RNA synthesis, were significantly decreased in the male pronuclei of H3.3R2A-EGFP-expressed zygotes compared with H3.3-EGFP-expressed and untreated zygotes (Figs. 3a, b and S7a). Furthermore, the signals for Pol II phosphorylated at Ser2 (Pol II Ser2P), which indicate elongation of Pol II, were significantly decreased in the H3.3R2A-EGFP-expressed zygotes (Figs. 3c, d and S7b), while pan-Pol II signals were equally detected in the H3.3R2A-EGFP-expressed zygotes compared with those in untreated zygotes (Fig. S7c–e). Interestingly, the localisation of pan-Pol II was significantly accumulated under the forced expression of H3.3-EGFP compared to that in the others (Fig. S7c–e). We next validated the expression levels of *MuERV-L*, which is one of the earliest transcribed retrotransposon using RT-PCR analysis^11,31^. The expression of *MuERV-L* was clearly downregulated in H3.3R2A-EGFP-expressed early 2-cell (24 hpi) embryos, which is the stage just before major ZGA (Figs. 4 and S8). These results suggest that methylation of H3.3R2 is involved in global transcription via activation of Pol II during minor ZGA.

### Formation of H3R2me2s, catalysed by PRMT5 and PRMT7, is associated with minor ZGA

**Figure 5:**
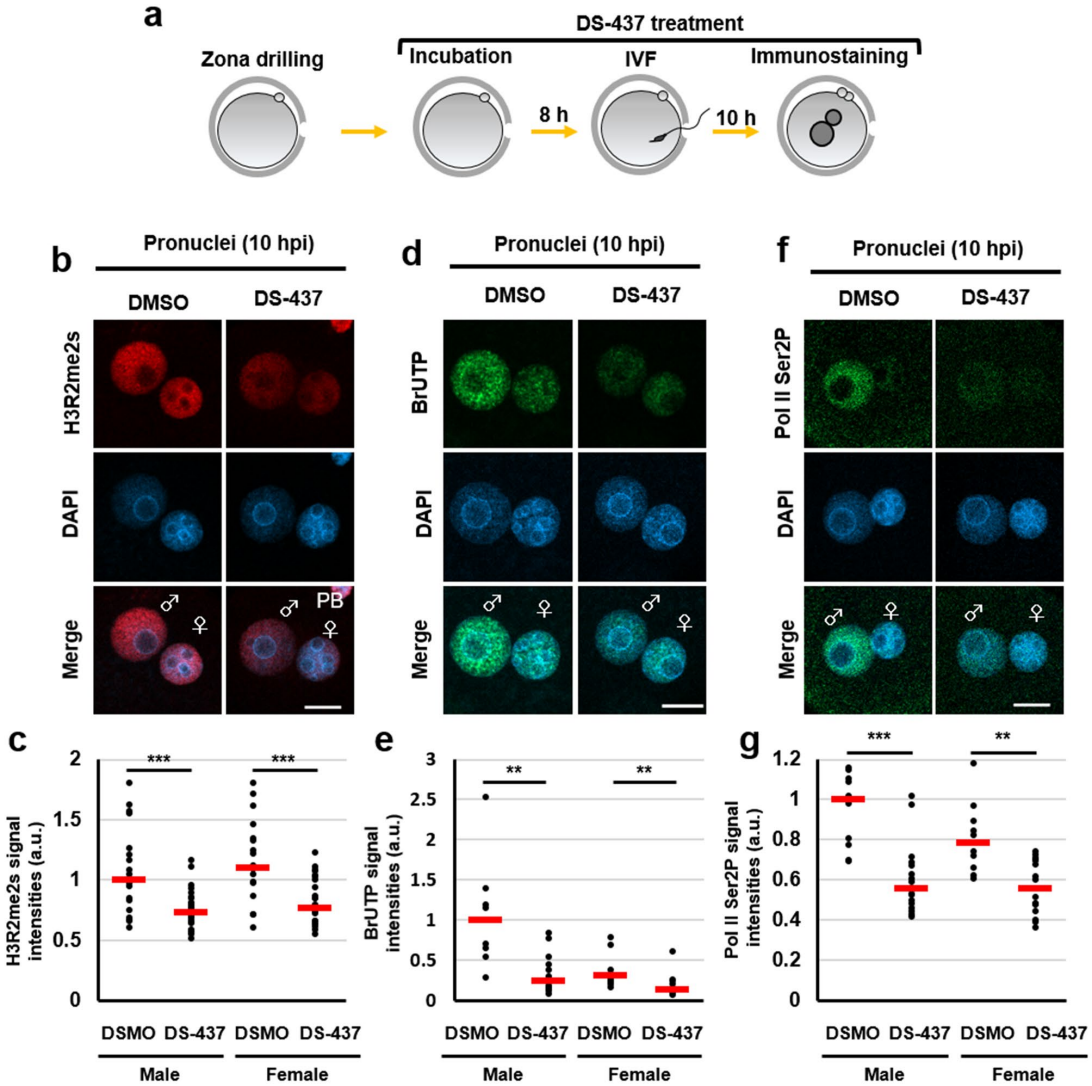
Reduction of transcriptional activities by DS-437-treatment in zygotes. (**a**) Schematic diagram of the experimental procedures. (**b**), (**d**), (**f**) Immunofluorescent images of H3R2me2s (red), BrUTP (green), Pol II Ser2P (green), and DAPI (blue) in DMSO- and DS-437-treated zygotes. DMSO treatment was used as a control. (**c**), (**e**), (**g**) Quantification of the H3R2me2s, BrUTP, and Pol II Ser2P signal intensities in pronuclei of DMSO- and DS-437-treated zygotes. Red bars indicate the median values. The median value of male pronuclei in untreated zygotes was set as 1. Mann–Whitney U test were performed to evaluate statistical significance. The number of zygotes in (**c**) was 14 for the DMSO-treated group and 28 for the DS-437-treated group. The number of zygotes in (**e**) was 9 for the DMSO-treated group and 12 for the DS-437-treated group. The number of zygotes in (**g**) was 10 for the DMSO-treated group and 16 for the DS-437-treated group. The pronucleus used for quantification are presented in Fig. S9.

**Figure 6:**
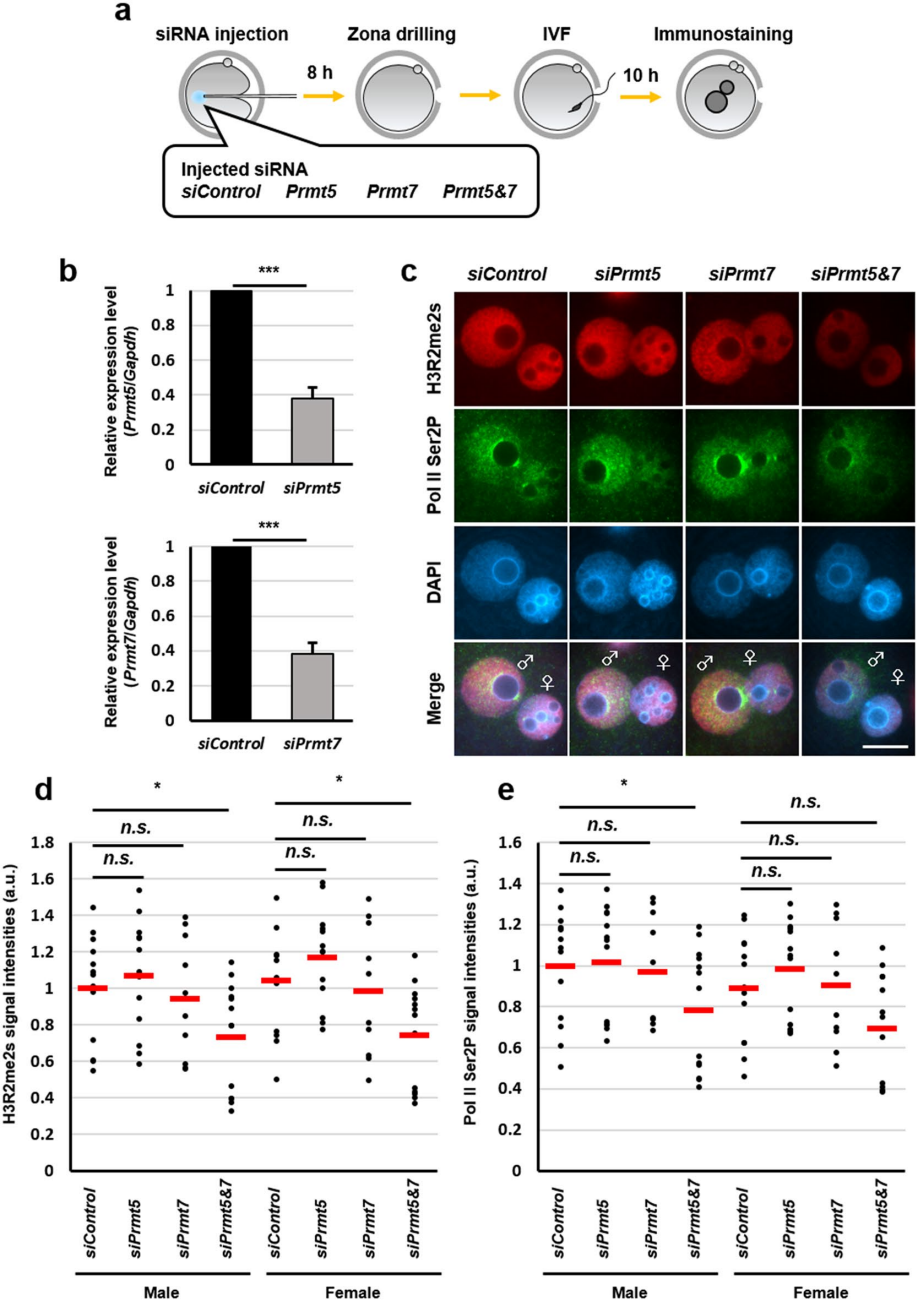
Knockdown of *Prmt5* and *Prmt7* mRNA revealed reduction in global transcription activities during minor ZGA. (**a**) Schematic diagram of the experimental procedures. (**b**) Knockdown of *Prmt5* and *Prmt7* expression by an antisense RNA injection was confirmed by quantitative RT-PCR analysis. The relative ratios were obtained by dividing the expression level of the *Prmt5* and *Prmt7* with the expression level of the *Gapdh*. Student’s t-test were performed for gel images. *Significantly different from the control (***p < 0.001). Bars represent the standard error of the mean. Full-length gels are presented in Fig. S10a. (**c**) Immunofluorescent images of H3R2me2s (red), Pol II Ser2P (green), and DAPI (blue) in *Prmt5* and *Prmt7* knockdown zygotes. *siControl* RNA was used as a control. (**d**), (**e**) Quantification of the H3R2me2s and Pol II Ser2P signal intensities in pronuclei of *Prmt5* and *Prmt7* knockdown zygotes. Dunnet test were performed to evaluate statistical significance. The number of zygotes in (**d**), (**e**) was 13 for the *siControl* group, 13 for the *siPrmt5* group, 10 for the *siPrmt7* group, and 13 for the *siPrmt5&7* group. The pronucleus used for quantification are presented in Fig. S10b.

A previous report showed that PRMT5 and PRMT7 are abundantly expressed in MII oocytes and preimplantation embryos by proteomic analyses^20^. First, to confirm whether endogenous H3R2me2s is catalysed by PRMT5 and PRMT7, we used the PRMT5–PRMT7 dual inhibitor DS-437. Because H3R2me2s signals were already observed in MII oocytes (Fig. 2a), we treated MII oocytes with DS-437 to interfere with de novo symmetric dimethylation of H3R2 (Fig. 5a). H3R2me2s signals were significantly decreased in male and female pronuclei of DS-437-treated zygotes compared with DMSO-treated zygotes as a control (Figs. 5b, c and S9a). These results indicate that endogenous H3R2me2s is reduced by DS-437 treatment during zygote development. Next, we examined the effect of DS-437 treatment on global RNA synthesis in zygotes at minor ZGA. The treatment of DS-437 resulted in the impairment of BrUTP incorporation and decreased Pol II Ser2P signals, particularly in male pronuclei (Figs. 5d–g and S9b, c). Finally, we attempted to determine the arginine methyltransferases that are important for the symmetrical dimethylation of H3R2 in zygotes. To this purpose, each *Prmt5* and *Prmt7* siRNA was injected into MII oocytes (Fig. 6a), and knockdown oocytes were obtained (Fig. 6b). A decrease in H3R2me2s and Pol II Ser2P signals was only observed by co-injection of *Prmt5* and *Prmt7* siRNAs (Figs. 6c–e, and S10), suggesting that both PRMT5 and PRMT7 catalyse H3R2me2s and are involved in Pol II activation in zygotes. To summarize, with the experiment on forced expression of H3.3R2A, these results suggest that symmetric dimethylation of H3R2 which are catalysed by both of PRMT5 and PRMT7 promotes transcriptional activity during minor ZGA.

### H3K4 methylation associates with H3R2me2s accumulation in male pronuclei at minor ZGA

**Figure 7:**
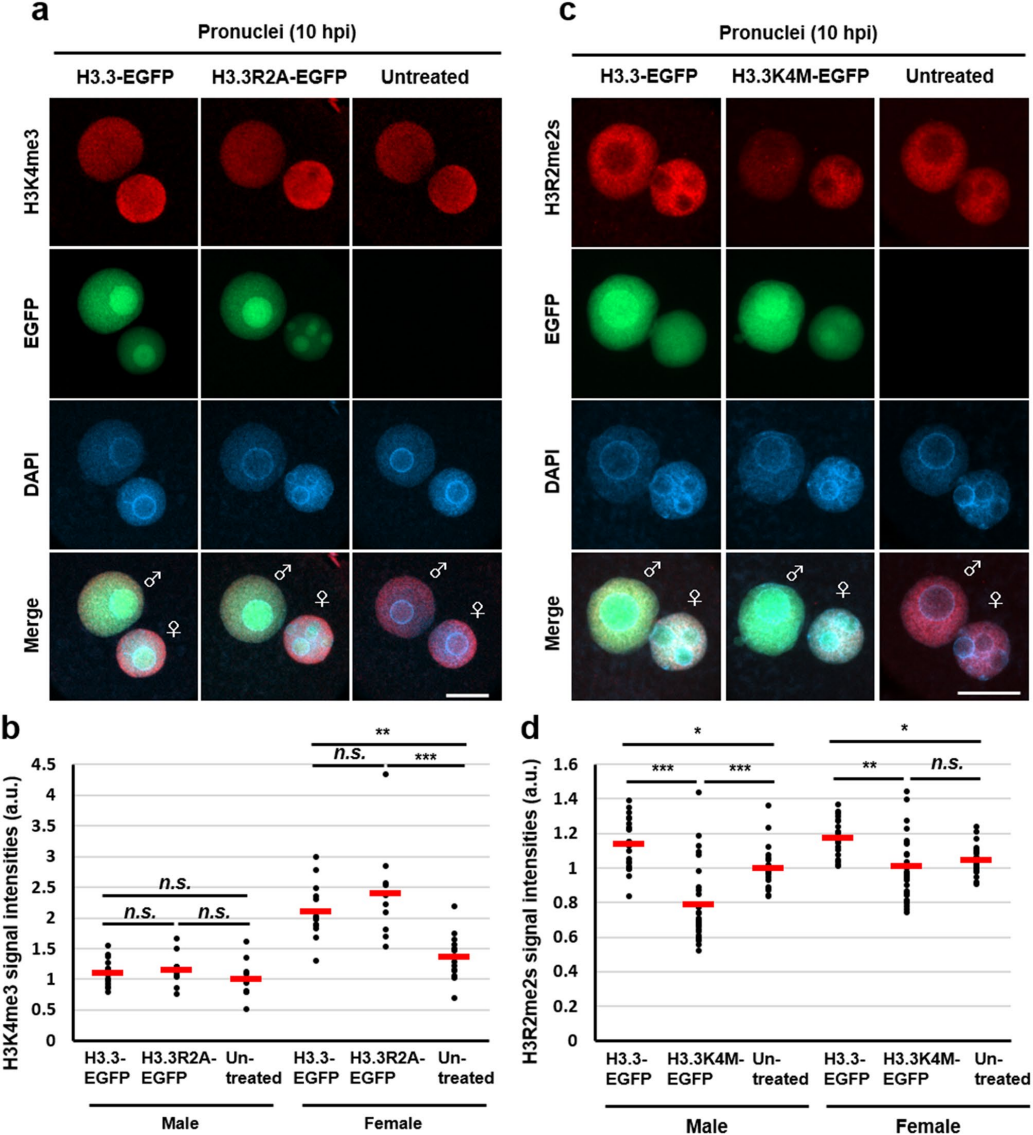
Expression of H3.3K4M mutant impaired H3R2me2s accumulation. (**a**), (**c**) Immunofluorescent images of H3K4me3 (red) or H3R2me2s (red) and DAPI (blue) in H3.3-EGFP-expressed, H3.3R2A-EGFP-expressed, H3.3K4M-EGFP-expressed, and untreated zygotes. EGFP (green) shows successful injection. Key: ♂, male pronuclei; ♀, female pronuclei. Scale bar = 20 µm. (**b**), (**d**) Quantification of the H3K4me3 and H3R2me2s signal intensities in pronuclei. Red bars indicate the mean values. The mean value of male pronuclei in untreated zygotes was set as 1. One-way ANOVA and the Tukey–Kramer test were performed to evaluate statistical significance. *Significantly different from the control (**p < 0.01, ***p < 0.001); *n.s.*: Not significantly different from the control. The number of zygotes in (**b**) was 15 for the H3.3-EGFP expressed group, 10 for the H3.3R2A-EGFP expressed group, and 13 for the untreated group. The pronucleus used for quantification are presented in Fig. S11a. The number of zygotes in (**d**) was 22 for the H3.3-EGFP expressed group, 29 for the H3.3K4M-EGFP-expressed group, and 23 for the untreated group. The pronucleus used for quantification are presented in Fig. S13.

A previous report showed that methylation of H3K4 in male pronuclei is required for minor ZGA in early mouse embryo^12^. In addition, H3K4me3 and H3R2me2s co-exist at transcription start sites in mouse B cells^32^. The H3K4me3 signal appears in male pronuclei at late PN stage (PN4)^12,24,33^, whereas our results showed that H3R2me2s was detected during all PN stages and was implicated in minor ZGA. These results prompted us to examine whether H3R2me2s is involved in H3K4me3 accumulation during minor ZGA. To show the relationship between H3K4me3 and H3R2me2s, we first investigated the effect of inhibiting H3R2me2s on H3K4me3 accumulation in zygotes at the PN4 stage by expressing H3.3R2A-EGFP and treating with DS-437. Paternal H3K4me3 signals showed no difference between either of the H3R2me2s inhibited zygotes and the control zygotes (Figs. 7a, b and S11a–d). However, H3K4me3 signals were significantly increased in female pronuclei of H3.3-EGFP and H3.3R2A-EGFP expressed zygotes compared with an untreated control (Figs. 7a, b and S11a). These results suggest that H3K4me3 deposition does not rely on H3R2me2s accumulation in male pronuclei. We next investigated the effect of preventing H3K4 methylation on H3R2me2s by forced expression of H3.3K4M-EGFP. We confirmed the expression of H3.3K4M-EGFP in MII oocytes and its localisation at the pronuclei of zygotes (Fig. S12a, b). Additionally, H3K4me3 signals were clearly diminished in male pronuclei, consistent with a previous report (Fig. S12c)^12^. Interestingly, the deposition of H3.3K4M caused a decrease in H3R2me2s signals in male pronuclei (Figs. 7c, d, and S13). These results suggest that H3R2me2s relies on H3K4 methylation in zygotes.

## Discussion

After fertilisation, protamines packaged around the sperm genome are immediately replaced with maternal histones, and the male genome undergoes chromatin remodelling for a transcriptionally active regions^34^. Here, we suggested that the formation of H3R2me2s, catalysed by PRMT5 and PRMT7, in the male genome is important for the first wave of transcription during zygote development. Generally, H3R2me2s is reported as a transcriptionally active marker^21,32^. Consistent with this, H3R2me2s incorporated into the male genome is detected in euchromatic regions during zygote development (Fig. 2). In addition, we have recently shown that H3R17me2a is associated with protamine–histone exchange and active DNA demethylation in zygotes^16^. These data suggest that histone arginine methylations in the paternal genome play an indispensable role for initial reprogramming events in zygotes.

Endogenous H3R2me2s was detected on maternal chromosomes and in female pronuclei in oocytes and zygotes, respectively (Fig. 2a). In female pronuclei, the efficient reduction of H3R2me2s signals at late PN stages was not observed, although the expressed signals of the unmethylatable mutant H3.3R2A-EGFP were clearly detected (Figs. 1d, e and S5). It is known that maternal chromatin largely maintains its histone methylations before and after fertilisation^3,35,36^. Indeed, endogenous H3R2me2s was clearly detected on the chromosomes in MII oocytes and was constantly observed throughout the PN stages (Fig. 2a). Thus, for reducing H3R2me2s in the female genome, the histone mutant would need to be expressed at an earlier stage during oocyte maturation. H3.3R2A-EGFP-expression can also prevent H3R2me2a acquisition, and it is possible that the interruption would result in the impairment of minor ZGA and 2-cell arrest in the H3.3R2A-EGFP-expressed embryos. H3R2me2a was detected at low levels in a part of the centromere region compared with H3R2me2s localised in the euchromatic regions (Fig. S6). In general, euchromatin allows gene expression, whereas heterochromatin, which is a highly compacted state, is transcriptionally repressive. Thus, based on immunofluorescent staining at the late PN stage, H3R2me2s would be associated with global transcription during minor ZGA rather than H3R2me2a in zygotes. Consistent with this hypothesis, the inhibition of PRMT5 and PRMT7, which catalyse symmetric dimethylation, using a dual inhibitor and their siRNAs resulted in a decrease of transcriptional activity in zygotes (Figs. 5d–g and 6c, e). In the previous report, inhibiting Pol II elongation by DRB treatment at the late 1-cell stage led to downregulation of the M-phase promoting genes, resulting in developmental arrest at the 2-cell stage^31^, suggesting that these gene expressions might be downregulated by PRMT5 and PRMT7 inhibition. However, we could not exclude the possibility of H3R2me2a involvement in early embryonic arrest at the 2-cell stage. Further studies, such as knockdown experiments of PRMT6, which catalyses H3R2me2a, are required to clarify these functions in early embryonic development. Interestingly, the dual inhibitor had more effect on BrUTP level than H3.3R2A forced expression; however, reduction in H3R2me2s levels was similar under both the conditions (Figs. 3a, b, and 5d, e), suggesting the possibility that PRMT5 and PRMT7 have other targets for transcription.

Although Pol II Ser2P accumulated at the same level between untreated and H3.3-EGFP-expressed zygotes (Fig. 3c–d), pan-Pol II signals were significantly increased under the forced expression of H3.3-EGFP compared with those in untreated and H3.3R2A-EGFP-expressed zygotes (Fig. S7c–e). According to previous reports, both H3R2me2s and H3K4me3 are recognised by WDR5, which is a component of the MLL complex, and these proteins interact with the Pol II complex^21,37^, suggesting that these modifications on H3.3-EGFP might contribute to the excessive recruitment of Pol II into the pronucleus. Another report indicates that excess H3 by the forced expression is not associated with chromatin^38^. Indeed, the global RNA levels and Pol II Ser2P were not different between untreated and H3.3-EGFP-expressed zygotes (Fig. 3a–d). Thus, there would be no effect of the global transcription activity, even though H3R2me2s accumulation was significantly increased in H3.3-EGFP-expression zygotes (Fig. 1d–e).

We hypothesised that H3R2me2s might regulate H3K4 methylation in mouse zygotes, since H3R2me2s and H3K4me3 are co-localised on the genome of mouse B cells^32^. However, our results showed that the forced expression of H3.3K4M results in a decrease of H3R2me2s level in male pronuclei (Fig. 7c, d), whereas H3.3R2A overexpression did not alter the level of H3K4me3 (Fig. 7a, c). These results suggest that the deposition of H3.3K4 methylation is related to H3R2me2s accumulation. Some studies have showed that H3K4me1 starts to localise at male pronuclei in PN1-2 zygotes^24^ and double knockdown of *Mll3* and *Mll4,* which are methyltransferases of H3K4me1, resulting in impairment of minor ZGA^12^. Conversely, accumulation of H3K4me3 in male pronuclei occurs from the PN4 stage onward^12,24^. Thus, there might be a zygote-specific relationship between H3K4me1 and H3R2me2s during minor ZGA. Since target genes for H3R2me2s have still not been reported, additional studies such as RNA-seq analyses would be required to uncover which genes are regulated by H3R2me2s.

The forced expression of H3.3-EGFP and H3.3R2A-EGFP resulted in an increase of maternal H3K4me3 compared with an untreated control at the late PN stage (Fig. 7a, b). In general, maternal H3K4me3 signals are clearly detected throughout zygote development^24,33^. Exogenous H3.3 is incorporated in female pronuclei later than in male pronuclei^24^. Therefore, we might detect the H3K4me3 signals of both chromatin incorporated endogenous H3 and free H3.3-EGFP and H3.3R2A-EGFP in female pronuclei. Because endogenous H3K4me3 accumulation in male pronuclei starts from the PN4 stage^24,33^, paternal H3K4me3 signals in these forced expression zygotes might not be increased at this stage.

The peptidylarginine deiminase (PADI) family positively converts charged arginine residues to citrulline and is associated with gene activation^39–41^. Recently, studies on early embryos have shown that the inhibition of PADI results in developmental arrest at the 2- or 4-cell stage^42^, and the loss of PADI1 leads to the impairment of genome activation at the 2-cell stage^43^. Interestingly, nuclear PADI1 is observed from the 2-cell stage but not the 1-cell stage^43^. These results might suggest that a genome activation mechanism at the 2-cell stage is required for the citrullinisation of histone arginine methylation unlike in the zygote. However, in the zygote and 2-cell embryo, the function of another histone arginine methylation is still unclear, and additional study is required to uncover the mechanisms by knockout of *Prmt* and *Padi* family.

In conclusion, our study reported that paternal H3R2me2s promotes the phosphorylation of Pol II Ser2 and RNA synthesis on the paternal genome during minor ZGA in mouse zygotes. Our results will contribute to elucidating the relationship between epigenetic modifications and transcriptional dynamics in zygotes.

## Materials and methods

### Animals

Mice (ICR strain) were purchased from Kiwa Laboratory Animals Co., Ltd. (Wakayama, Japan) and Japan SLC, Inc. (Shizuoka, Japan) and were maintained in light-controlled, air-conditioned rooms. This study was performed in strict accordance with the ARRIVE guidelines and the Fundamental Guidelines for Proper Conduct of Animal Experiment and Related Activities in Academic Research Institutions under the jurisdiction of the Ministry of Education, Culture, Sports, Science and Technology of Japan and the Guidelines of Kindai University and Kyoto University for the Care and Use of Laboratory Animals. The protocol was approved by the Committee on the Ethics of Animal experiments of Kindai University (Permit Number: KABT-26–002 and KABT-26–003) and Kyoto University (Permit Number: Med Kyo 19,587). All mice were killed by cervical dislocation, and all efforts were made to minimise suffering and to reduce the number of animals used.

### Collection of spermatozoa and oocytes

Collection of spermatozoa and oocytes was performed as described in previous studies^44–52^. Spermatozoa were collected from the cauda epididymis of male mice. The sperm suspension was incubated in HTF medium (Ark Resource Co., Ltd., Kumamoto, Japan) for 1–1.5 h to allow for capacitation at 37 °C under 5% CO_2_ in air. Oocytes were collected from the excised oviducts of female mice (2–3 months old) that were superovulated with pregnant mare serum gonadotropin (PMSG; Serotropin, Teikoku Zoki, Tokyo, Japan) and 48 h later with human chorionic gonadotropin (hCG; Puberogen, Sankyo, Tokyo, Japan). Cumulus-oocyte complexes were recovered into pre-equilibrated HTF medium.

### In vitro fertilisation (IVF) and embryo culture

The sperm suspension was added to the cumulus-oocyte complex cultures and morphologically normal zygotes were collected at 2 h post insemination (hpi). The zygotes were cultured in potassium simplex optimised medium (KSOM) (Ark Resource) at 37 °C under 5% CO_2_ in air.

### Plasmid construction and in vitro mRNA transcription

Each cDNA for H2AX-EGFP, H3.3-EGFP, and H4-EGFP was cloned from an ovary cDNA library using a pUC118 vector (Takara Bio Inc., Shiga, Japan; 3318) containing the *egfp* sequence. Each cDNA for H2AXR3A-EGFP, H3.3R2A-EGFP, H3.3R8A-EGFP, H4R3A-EGFP and H3.3K4M-EGFP was cloned from the pUC118 vector containing each cDNA for H2AX-EGFP, H3.3-EGFP and H4-EGFP using mutagenesis primers (Table S3). These cDNAs were subcloned into a pcDNA3.1-poly (A) 83 vector^53^. In vitro transcription was performed using a MEGAscript T7 Transcription Kit (Thermo Fisher Scientific, Massachusetts, USA; AM1333, AM1334) and Cap Analog (m7G(5′)ppp(5′)G) (Thermo Fisher Scientific; AM8048).

### Microinjection of mRNA or siRNA into MII oocytes

Approximately 3–5 pL of mRNA or siRNA was microinjected into the cytoplasm of MII oocytes in M2 medium (Ark Resource) using an inverted microscope (IX70 and IX71, Olympus, Tokyo, Japan) equipped with a Piezo impact drive unit (Prime Tech Ltd., Ibaraki, Japan) and a micromanipulator (Narishige, Tokyo, Japan). For the translation of the mRNA, the microinjected MII oocytes were incubated at 37 °C under 5% CO_2_ in air for 4 h in M2 medium (pH = 7.4) or HTF medium as described in the previous report^12^. For knockdown of *Prmt5* and/or *Prmt7* mRNA, 25 µM siRNA-microinjected MII oocytes were incubated at 37 °C under 5% CO_2_ in air for 8 h in HTF medium in the previous report^49^. The siRNAs used were Silencer Select Predesigned siRNAs (Thermo Fisher Scientific). Their purchase IDs are *Prmt5*, s77695; *Prmt7*, s103119. As a *siControl,* 25 µM of Silencer Negative Control No. 1 siRNA (Thermo Fisher Scientific; AM4611) was used. Following incubation, the successful mRNA-injection into MII oocytes was confirmed by observing EGFP signals, using the inverted microscope equipped with a mercury lamp (U-RFL-T, Olympus) and a digital CCD camera (DP72 and DP73, Olympus).

### Zona drilling

To fertilise cumulus cells-removed oocytes, a part of the zona pellucida was drilled by using a Piezo impact drive unit (Prime Tech Ltd.) in M2 medium or using laser equipment (XYClone, Hamilton Thorne Biosciences, Beverly, MA, USA) in 0.5 M sucrose/M2 medium. Laser perforation was performed as described in a previous study^54^. Briefly, the laser was applied to the point on the zona pellucid that showed the widest perivitelline space, and a hole (approximately 6 µm) was perforated in each zygote (wavelength 1,460 nm; output 300 mW; pulse width 120 µs). The oocytes were washed and inseminated in HTF medium and morphologically normal zygotes were collected at 1 hpi. The zygotes were cultured in KSOM at 37 °C under 5% CO_2_ in air. Zona-drilled zygotes obtained by laser perforation were used only for the immunofluorescent staining analysis.

### DS-437 treatment for inhibition of PRMT5–PRMT7 activity

In a previous report, a PRMT5 and PRMT7 dual inhibitor, DS-437, effectively inhibited the methyltransferase activity at 100 µM^55^. Zona-drilled oocytes were moved into M2 medium (pH = 7.4) containing 100 µM DS-437 and incubated at 37 °C under 5% CO_2_ in air for 8 h. DS-437-treated oocytes were washed three times in HTF medium containing 100 µM DS-437, inseminated, and cultured in the medium.

### Global transcription assay

For the global transcription assay, 100 µM BrUTP (Sigma, St. Louis, MO, USA) was microinjected into zygotes at 8 hpi. After 2 h, BrUTP injected zygotes were used for immunofluorescent analysis.

### Immunofluorescent analysis

The classification of pronuclear (PN) stages was performed according to previous reports^56,57^, in which the pronuclear morphology and hpi were considered. Subcellular localisation of H3R2me2s was determined by the immunofluorescent analyses of zygotes, as described in previous studies^44,45,47,49–52,57^. Embryos were fixed in 4% paraformaldehyde (Nacalai Tesque, Kyoto, Japan) at RT for 15 min, washed three times in PBS containing 0.01% polyvinyl alcohol (PBS-PVA), and permeabilised with PBS containing 1% bovine serum albumin (PBS-BSA) and 0.5% Triton X-100 (Nacalai Tesque) at RT for 15 min. The embryos were then incubated with anti-H3R2me2s antibody (1:100; Abcam; ab194684), anti-H3R2me2a antibody (1:100; Abbexa; abx000029), anti-H3K4me3 antibody (1:100,000; Abcam; ab8580), Alexa Fluor 594 anti-RNA Polymerase II RPB1 Antibody (1:100; BioLegend; 664,908), anti-RNA polymerase II phosphorylated at Ser2 (Pol II Ser2P) antibody (1:1,000; BioLegend; 920,203 and 920,204), and BrdU antibody (1:100; Sigma; 11,170,376,001) in 1% PBS-BSA at 4 °C overnight. After incubation, the samples were washed three times in 1% PBS-BSA and treated for 1 h at RT with Alexa Fluor 594-labelled donkey anti-rabbit IgG secondary antibody (1:2,000; Life Technologies, Carlsbad, CA, USA; A-21207) for anti-H3R2me2s, H3R2me2a, H3K4me3, and BrdU antibody; with Alexa Fluor 488-labelled goat anti-mouse IgG secondary antibody (1:2,000; Invitrogen, Carlsbad, CA, USA; A-11001) for anti-BrdU antibody; or with Alexa Fluor 594-labelled donkey anti-mouse IgM secondary antibody (1:2,000; Invitrogen; A-21044) or Alexa Fluor 488-labelled donkey anti-mouse IgM secondary antibody (1:2,000; Invitrogen; A-21042) for anti-Pol II Ser2P antibody. Stained zygotes were mounted on glass slides in a Vectashield mounting medium (Vector Laboratories, Burlingame, CA, USA) containing 3 µg/ml DAPI (Invitrogen; D1306). Finally, the H3R2me2s, Pol II Ser2P stained samples were imaged using a conventional upright microscope (Axioplan2, Carl Zeiss, Jene, Germany) equipped with a mercury lamp (HBO 100, Carl Zeiss) and digital CCD camera (AxioCamMRc5, Carl Zeiss), or BZ-X700 (Keyence, Osaka, Japan). The H3R2me2s-, H3R2me2a-, H3K4me3-, Pol II Ser2P-, pan-Pol II-, and BrUTP-stained samples were imaged using a confocal laser-scanning microscope (LSM 800, Carl Zeiss) equipped with 40 × and 63 × silicon oil-immersion objectives (Carl Zeiss).

### Image analysis

Images were analysed using ZEN imaging software 2.3 (Carl Zeiss) and ImageJ software (National Institutes of Health). To quantify the pronuclear signal of H3R2me2s, BrUTP, Pol II Ser2P, and H3K4me3, all focal planes that covered the pronuclear region were merged by average intensity projection, followed by selecting the whole PN, the average intensities were measured.

### RT-PCR analysis

Total RNA was isolated from 30 pooled oocytes and 20 pooled embryos followed by synthesis of cDNA using the RNeasy Plus Micro Kit (50) (QIAGEN; 74,034). A random 6 mer (Takara Bio Inc.; RR037A) was used as the first strand primer. Armoured RNA control provided from Cells-to-cDNA II Kit (Thermo Fisher Scientific; AM1723) as an external control was spiked into the cell lysate containing total RNA samples before the RT reaction. Half of the cell lysate without the RT enzyme was used as a negative control. cDNA was synthesised using the Superscript III RT First-Strand system (Life Technologies; 18,080,051). Following the RT reaction, the prepared cDNA samples were amplified and analysed using PCR. The primers used are described in Table S4. Amplifications were run in a Thermal Cycler Dice TP600 (Takara Bio Inc.) and Takara Taq Hot start version (Takara Bio Inc.; R007A). The reaction parameters were as follows: one cycle at 98 °C for 2 min and 40 cycles for *Armored RNA control* or 26 cycles for *MuERV-L* at 98 °C for 10 s, 58 °C for 30 s, and 72 °C for 25 s. The RT-PCR products were electrophoresed on 2% agarose gels with TBE and visualised using Midori Green Advanced (1:10,000; NIPPON Genetics Co., Ltd; NE-MG04). Three independent experiments were performed for each.

### Statistical analysis

We performed the Student’s t-test, chi-squared test, one-way ANOVA and Tukey–Kramer test, and Dunnett test with an α level of 0.05 to determine possible statistically significant differences.

## Supplementary Information

Supplementary Information 1.
