# Supplementary material for: Symmetrically dimethylated histone H3R2 promotes global transcription during minor zygotic genome activation in mouse pronuclei
Source: Sci Rep. 2021 May 12;11:10146. doi: 10.1038/s41598-021-89334-w (PMC8115239; doi:10.1038/s41598-021-89334-w)
Supplement: Supplementary file 1 — Supplementary Information 1. [file 41598_2021_89334_MOESM1_ESM.pdf]

Supplementary Information

Symmetrically dimethylated histone H3R2 promotes global transcription during minor zygotic genome activation in mouse pronuclei

Kohtaro Morita[1,2,*], Yuki Hatanaka[3,4], Shunya Ihashi[1], Masahide Asano[2], Kei Miyamoto[1] & Kazuya Matsumoto[1]

[1]Laboratory of Molecular Developmental Biology, Graduate School of Biology-Oriented Science and Technology, Kindai University, Wakayama, Japan.
[2] Institute of Laboratory Animals, Graduate School of Medicine, Kyoto University, Kyoto, Japan.
[3]RIKEN BioResource Research Center, Tsukuba, Ibaraki, Japan.
[4]Medical Research Council (MRC) London Institute of Clinical Sciences, Imperial College London, London, UK.

*Corresponding author
E-mail: morita.kohtaro.w84@kyoto-u.jp

Table S1. Examination of mRNA concentrations not affecting early embryonic development.

Concentration of *H3f3b-egfp* mRNA	No. of oocytes	No. of injected oocytes	No. (%) of surviving oocytes at the time of IVF	No. (%) of 2PN zygotes	No. (%) of EGFP (+) 2PN zygotes	No. (%) of embryos developed to				
						2-cell	4-cell	8-cell	Morula	Blastocyst
0 ng/μL (Untreated)	78	-	78 (100)	40 (51±6.8)	0 (0)	38 (95±2.0)	34 (85±1.1)	29 (73±5.3)	26 (65±7.7)	25 (63±8.2)[a]
50 ng/μL	108	108	108 (100)	50 (46±6.8)	50 (100)	47 (94±2.0)	45 (90±3.7)	35 (70±14.0)	33 (66±11.0)	28 (56±15.8)[a,b]
100 ng/μL	100	100	97 (97±1.8)	40 (41±0.9)	40 (100)	38 (95±1.7)	31 (78±4.7)	26 (65±3.5)	26 (65±3.5)	16 (40±14.8)[b]

Mean ± S.E. from three independent experiments.
Different superscripts indicate significant differences from the blastocyst stage in the control (untreated) ($p < 0.05$).

Table S2. Effects of mutant histone expression on early embryonic development.

Injected mRNA	No. of injected oocytes	No. (%) of surviving oocytes at the time of IVF	No. (%) of 2PN zygotes	No. (%) of EGFP (+) 2PN zygotes	No. (%) of embryos developed to				
					2-cell	4-cell	8-cell	Morula	Blastocyst
H2AX-EGFP	178	148 (83±2.2)	81 (55±12.1)	81 (100)	80 (99±0.7)[a]	80 (99±0.7)[a]	75 (93±1.4)[a]	71 (88±2.9)[a]	63 (78±3.2)[a]
H2AXR3A-EGFP	191	165 (86±3.2)	81 (49±7.4)	81 (100)	80 (99±1.0)[a]	76 (94±5.2)[a]	70 (86±5.4)[a]	69 (85±4.6)[a]	63 (78±6.8)[a]
H3.3-EGFP	190	176 (93±1.8)	78 (44±12.6)	78 (100)	76 (97±1.4)[a]	63 (81±5.6)[a]	51 (65±11.6)[a]	51 (65±11.6)[a]	46 (59±4.9)[a]
H3.3R2A-EGFP	217	198 (91±3.8)	82 (41±5.5)	82 (100)	74 (90±4.0)[a]	9 (11±6.8)[b]	5 (6±4.5)[b]	5 (6±4.5)[b]	4 (5±4.1)[b]
H3.3R8A-EGFP	189	177 (94±3.0)	74 (42±3.5)	74 (100)	72 (97±1.6)[a]	63 (85±2.0)[a]	58 (78±7.0)[a]	56 (76±5.7)[a]	50 (68±6.4)[a]
H4-EGFP	161	150 (93±3.7)	74 (49±4.2)	74 (100)	73 (99±1.0)[a]	70 (95±0.4)[a]	54 (73±1.6)[a]	54 (73±1.6)[a]	52 (70±1.8)[a]
H4R3A-EGFP	160	149 (93±2.8)	59 (40±4.1)	59 (100)	58 (98±1.3)[a]	57 (97±0.4)[a]	47 (80±4.1)[a]	47 (80±4.1)[a]	42 (71±7.0)[a]

Mean ± S.E. from three independent experiments.
Different superscripts indicate significant differences from the control (WT) ($p < 0.001$).

Table S3. Mutagenesis primers used in this study.

	Forward primers (5' to 3')	Reverse primers (5' to 3')
H2AXR3A-EGFP	GCCAAGCTTATGTCCGGAGCCGGC	GTTGAATTCGAGCTCGGTACCCAATTC
H3.3R2A-EGFP	AAGCTTATGGCCGCAACCAAGCAGACCGCTAGG	TCTGCTTGGTTGCGGCCATAAGCTTAACTAG
H3.3R8A-EGFP	ACCGCTGCGAAGTCCACCGGTGGGAAA	GGACTTCGCAGCGGTCTGCTTGGTTCG
H4R3A-EGFP	GCCAAGCTTATGTCGGGTGCCGGC	GTTGAATTCGAGCTCGGTACCCAATTC

Red font indicates an alanine codon.
Each cDNA for mutant histones was cloned from pUC118-containing *egfp*-tagged *H2ax*, *H3f3b*, or *H4* sequences.
cDNA for H3.3R2A-EGFP and H3.3R8A-EGFP was cloned by inverse PCR.

Table S4. RT-PCR primers used in this study.

	Forward primers (5' to 3')	Reverse primers (5' to 3')
Gapdh	AACTTTGGCATTGTGGAAGG	ACACATTGGGGGTAGGAACA
MuERV-L	TTCTCAAGGCCCACCAATAGT	GACACCTTTTTTAACTATGCGAGCT
Prmt5	GTAATTGAGAATGCCCCGAC	GGTTAGGAAAATGCTGGTGG
Prmt7	CAGTGTCATCAATAAACGGCC	CATCAAAACCTTCGCAGTCA

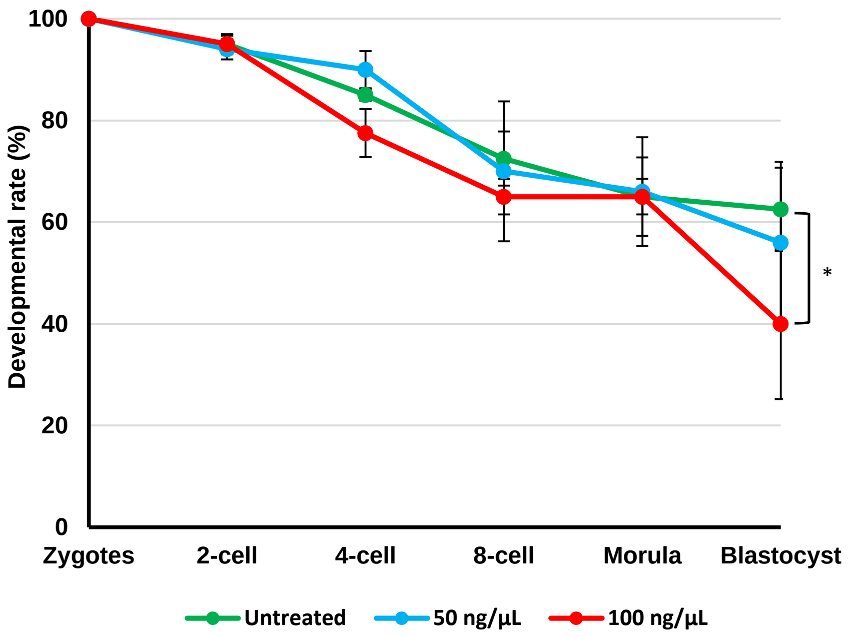

Fig. S1. Examination of conditions for expressing EGFP-tagged exogenous histones in early embryos. Developmental rates of embryos expressing H3.3-EGFP (*H3f3b-egfp* mRNA 50 ng/μL: blue bar, 100 ng/μL: red bar) and untreated (green bar) embryos. Mean ± S.E. from three independent experiments. A chi-squared test was performed for analysis of embryonic development until the blastocyst stage. An asterisk indicates statistical significance (*p < 0.05).

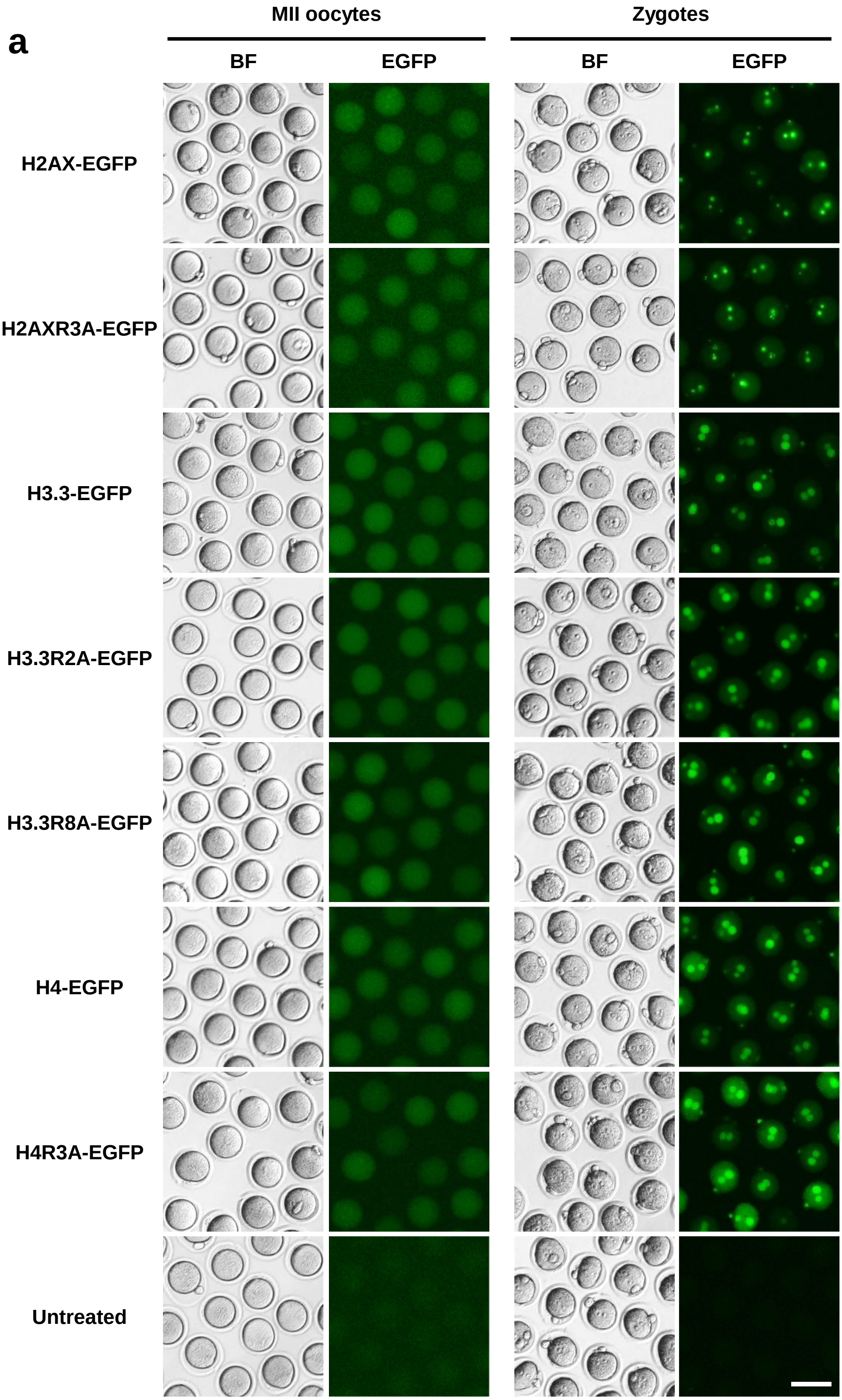
a
MII oocytes
Zygotes
BF
EGFP
BF
EGFP
H2AX-EGFP
H2AXR3A-EGFP
H3.3-EGFP
H3.3R2A-EGFP
H3.3R8A-EGFP
H4-EGFP
H4R3A-EGFP
Untreated

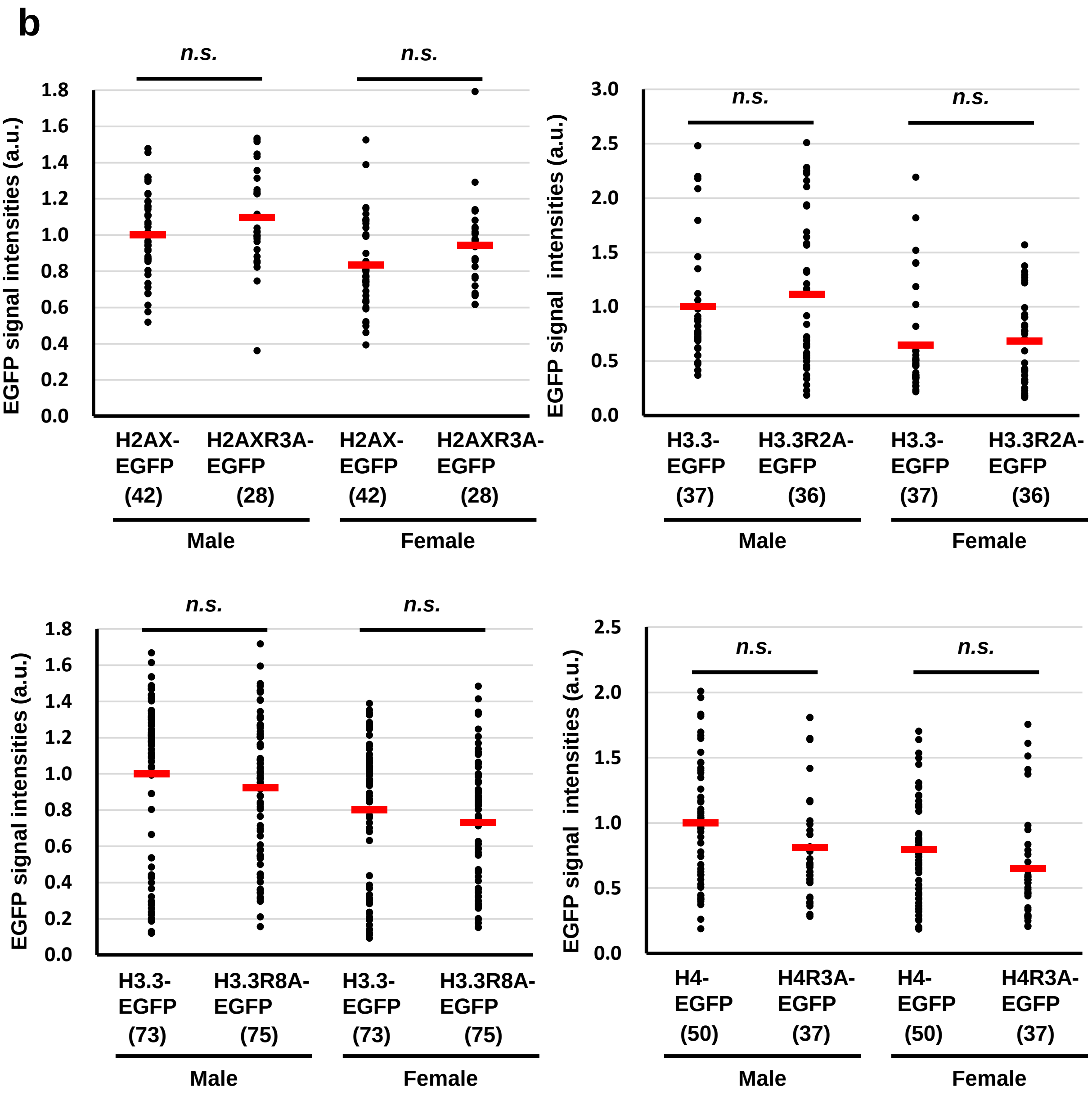

Fig. S2. Expression and localisation of EGFP-tagged exogenous histones in the cytoplasm of oocytes and pronuclei of zygotes. (a) Observation of EGFP-signals in each mRNA-injected oocyte at 4 h after injection and zygotes at 6 hpi from each mRNA-injected oocyte. Scale bar = 100 µm. (b) Quantification of EGFP signal intensities in the pronuclei of H2AX-EGFP, H2AXR3A-EGFP, H3.3-EGFP, H3.3R2A-EGFP, H3.3R8A-EGFP, H4-EGFP, or H4R3A-EGFP-expressed zygotes. Each dot plot represents a single zygote. Red bars indicate the mean values. The mean value of male pronuclei in each control zygote was set as 1. Student's t-test was performed to evaluate statistical significance. *n.s.*: Not significantly different from the control.

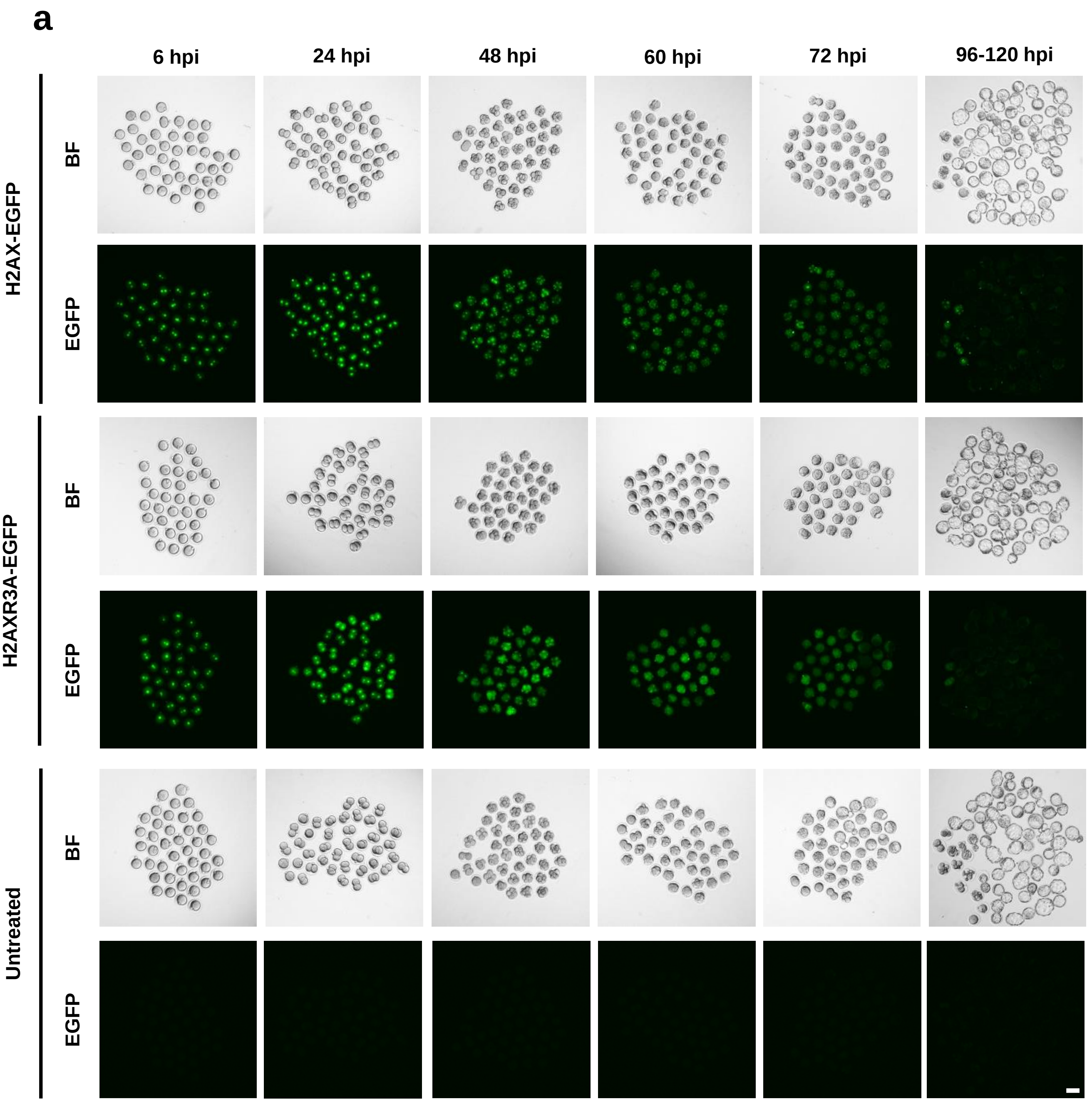
a
6 hpi
24 hpi
48 hpi
60 hpi
72 hpi
96-120 hpi
H2AX-EGFP
BF
EGFP
H2AXR3A-EGFP
BF
EGFP
Untreated
BF
EGFP

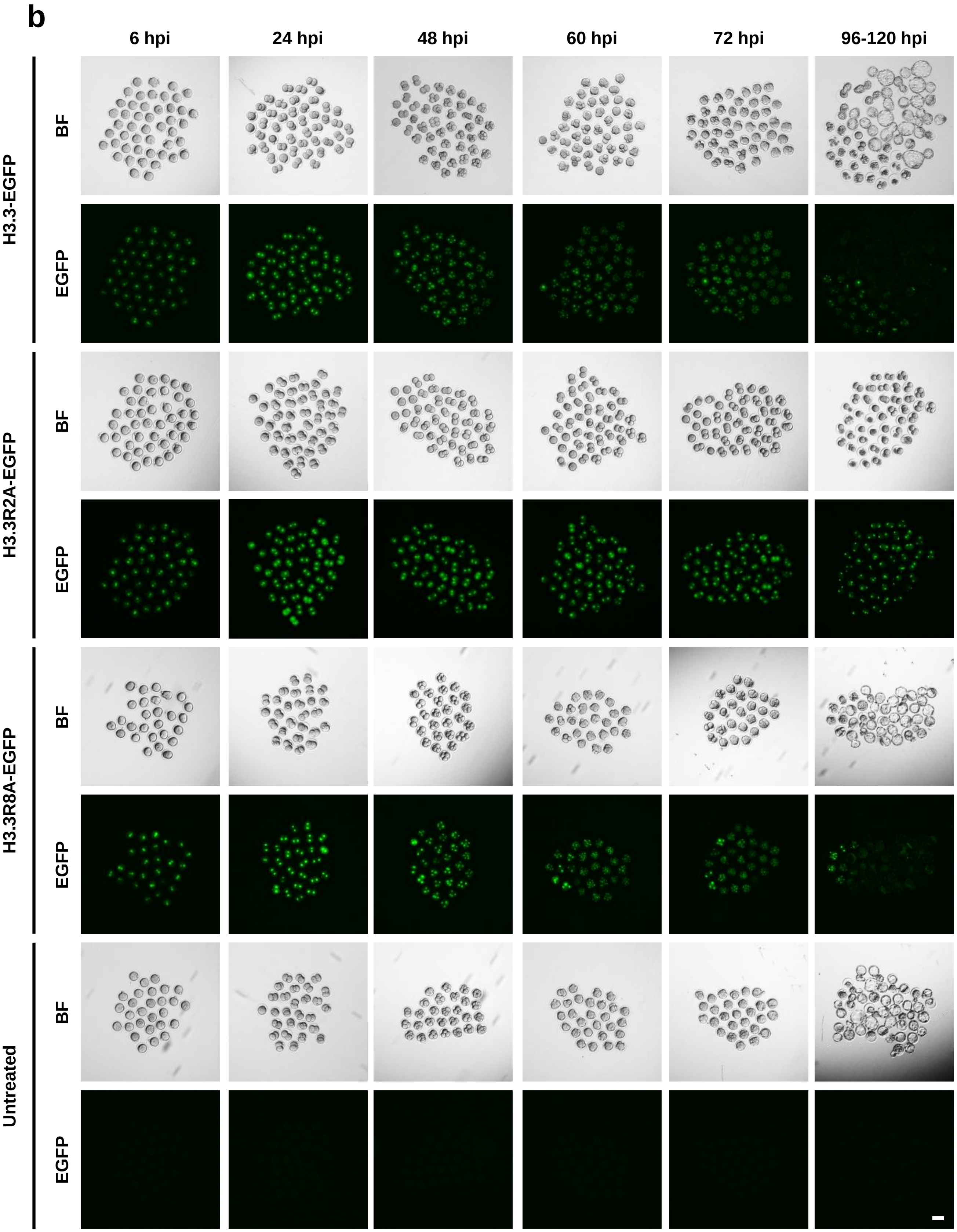

b
6 hpi
24 hpi
48 hpi
60 hpi
72 hpi
96-120 hpi
H3.3-EGFP
H3.3R2A-EGFP
H3.3R8A-EGFP
Untreated
BF
EGFP

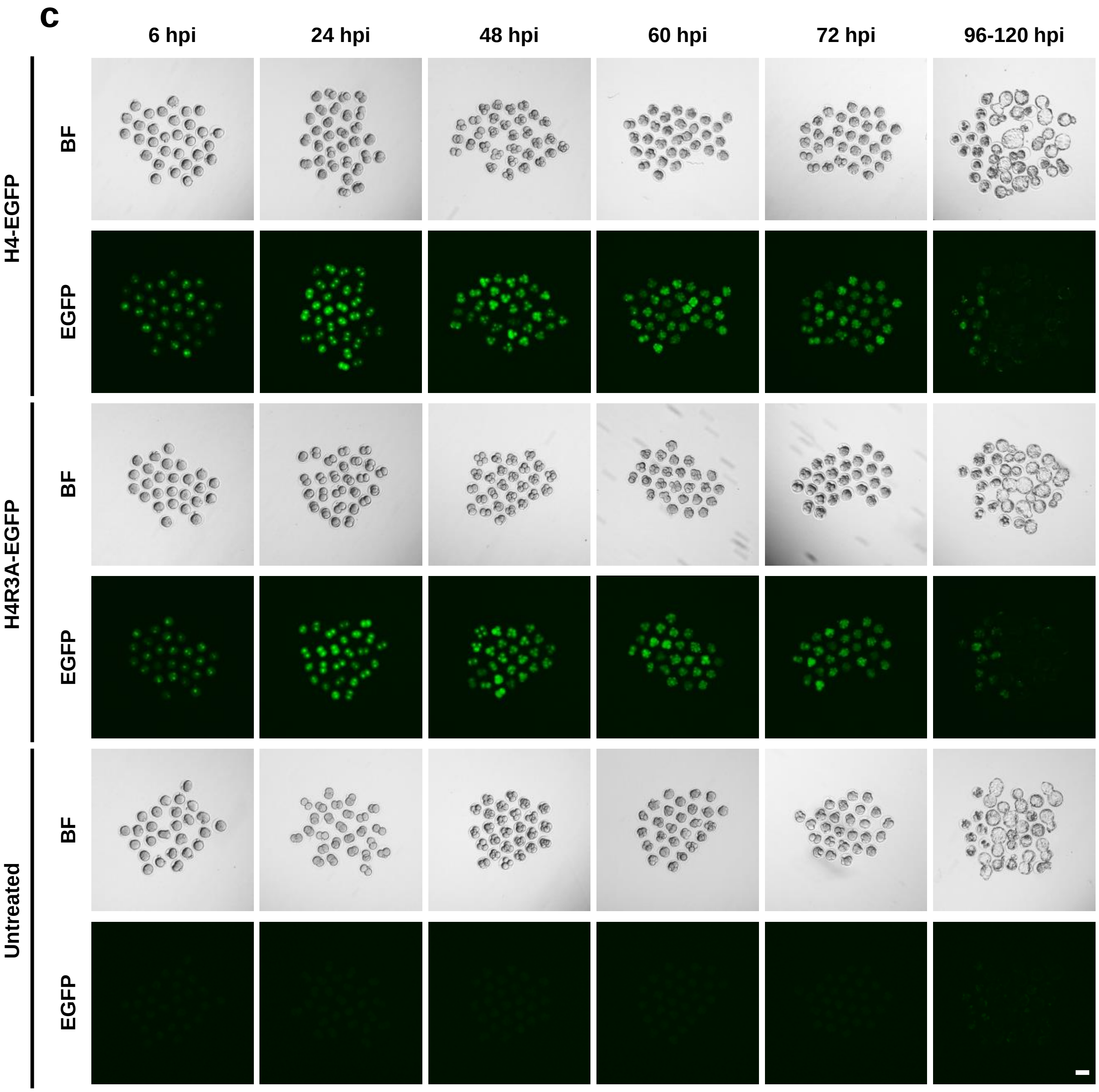

Fig. S3. Effects of dominant-negative mutations H2AXR3A, H3.3R2A, H3.3R8A, and H4R3A on embryonic development. (a–c) Representative images of each embryo during 6–120 hpi are shown. Scale bar = 100 µm.

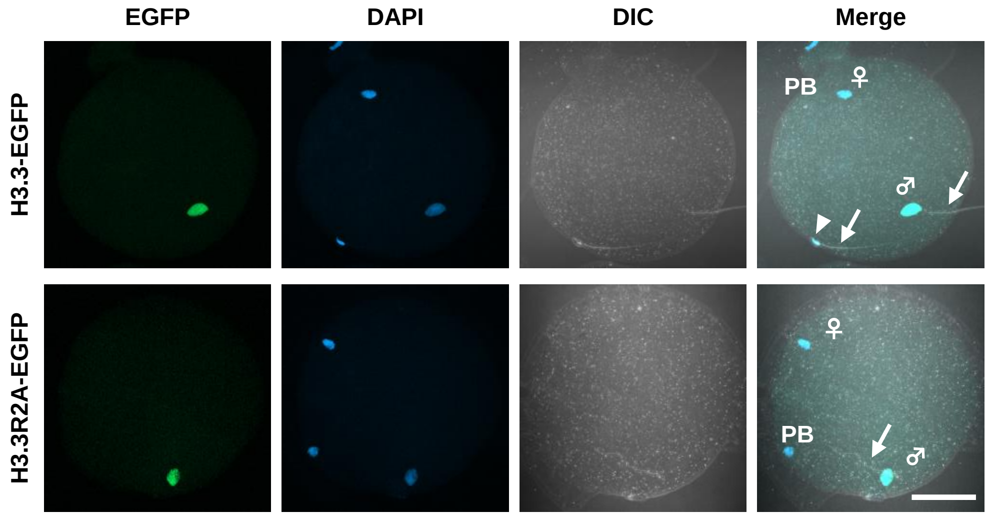

Fig. S4. H3.3-EGFP and H3.3R2A-EGFP were predominantly incorporated into male pronuclei. Representative images of H3.3-EGFP-expressed and H3.3R2A-EGFP-expressed zygotes at 2 hpi. Key: ♂, male pronuclei; ♀, female pronuclei; PB, polar body; arrowhead, sperm head; arrow, sperm tail. Scale bar = 20 µm.

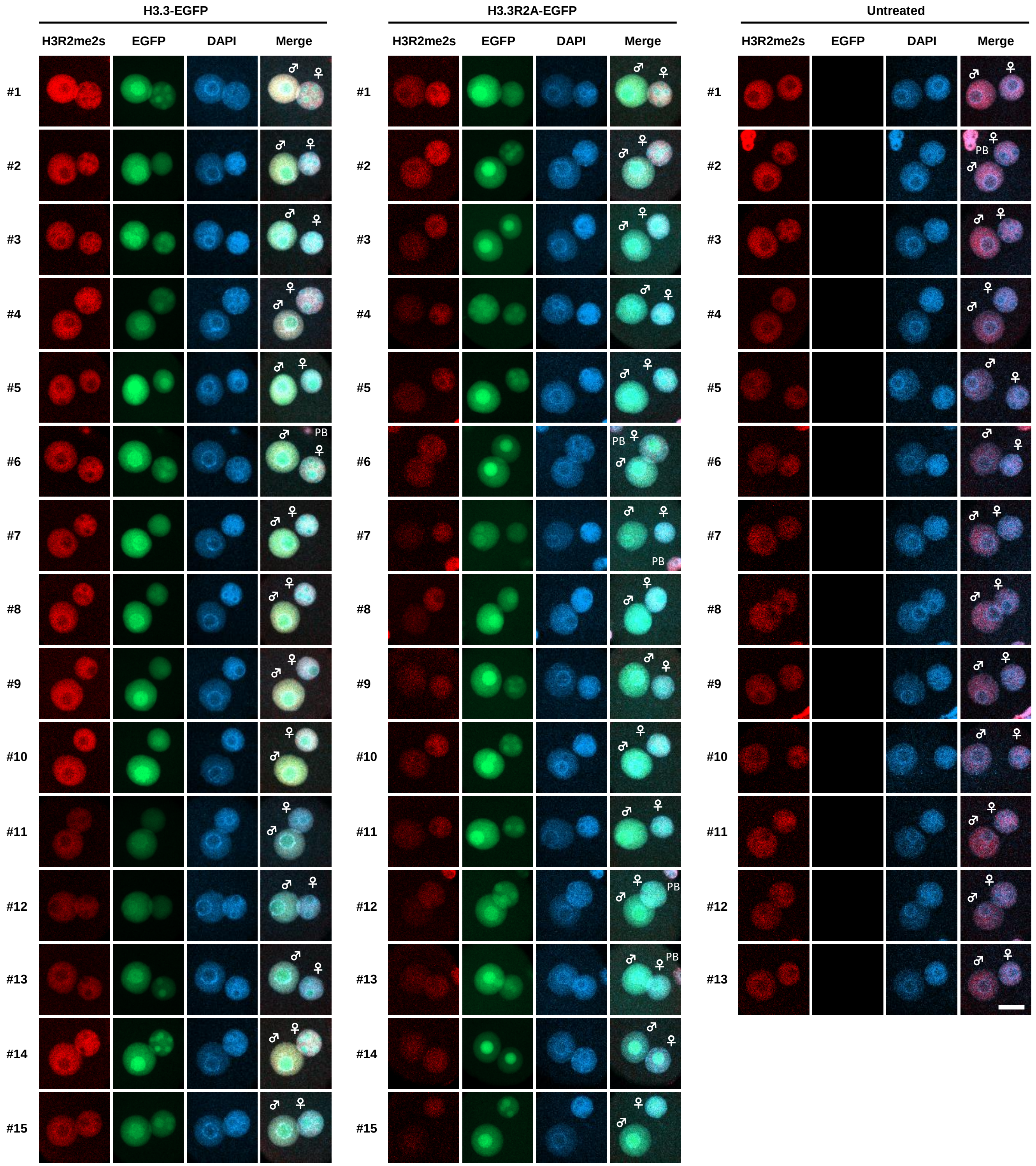

Fig. S5. Images of H3R2me2s staining in H3.3-EGFP-expressed, H3.3R2A-EGFP-expressed, and untreated zygotes at 10 hpi. Images of H3R2me2s (red)-stained zygotes for Fig. 1e. EGFP (green) shows successful injection. DAPI is indicated as DNA. Key: ♂, male pronuclei; ♀, female pronuclei; PB, polar body. Scale bar = 20 μm.

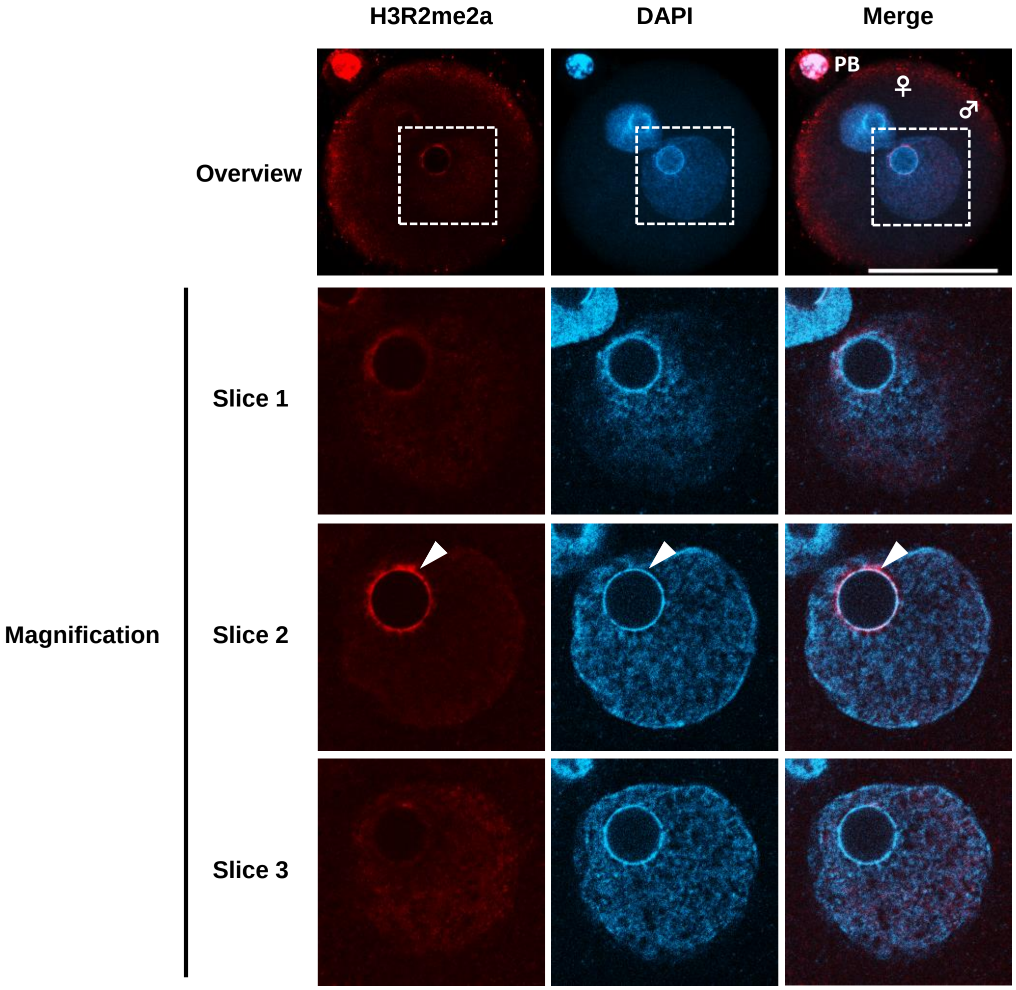

Fig. S6. Localisation of H3R2me2a in pronuclei of zygotes. Immunostaining for localisation of H3R2me2a in PN5 stage zygotes. The representative images of zygotes stained with DAPI (blue) and anti-H3R2me2a antibody (red). Male pronuclei corresponding to dashed squares are shown magnified below. Key: ♂, male pronuclei; ♀, female pronuclei; PB, polar body; arrowhead, sperm. Scale bar = 50 µm.

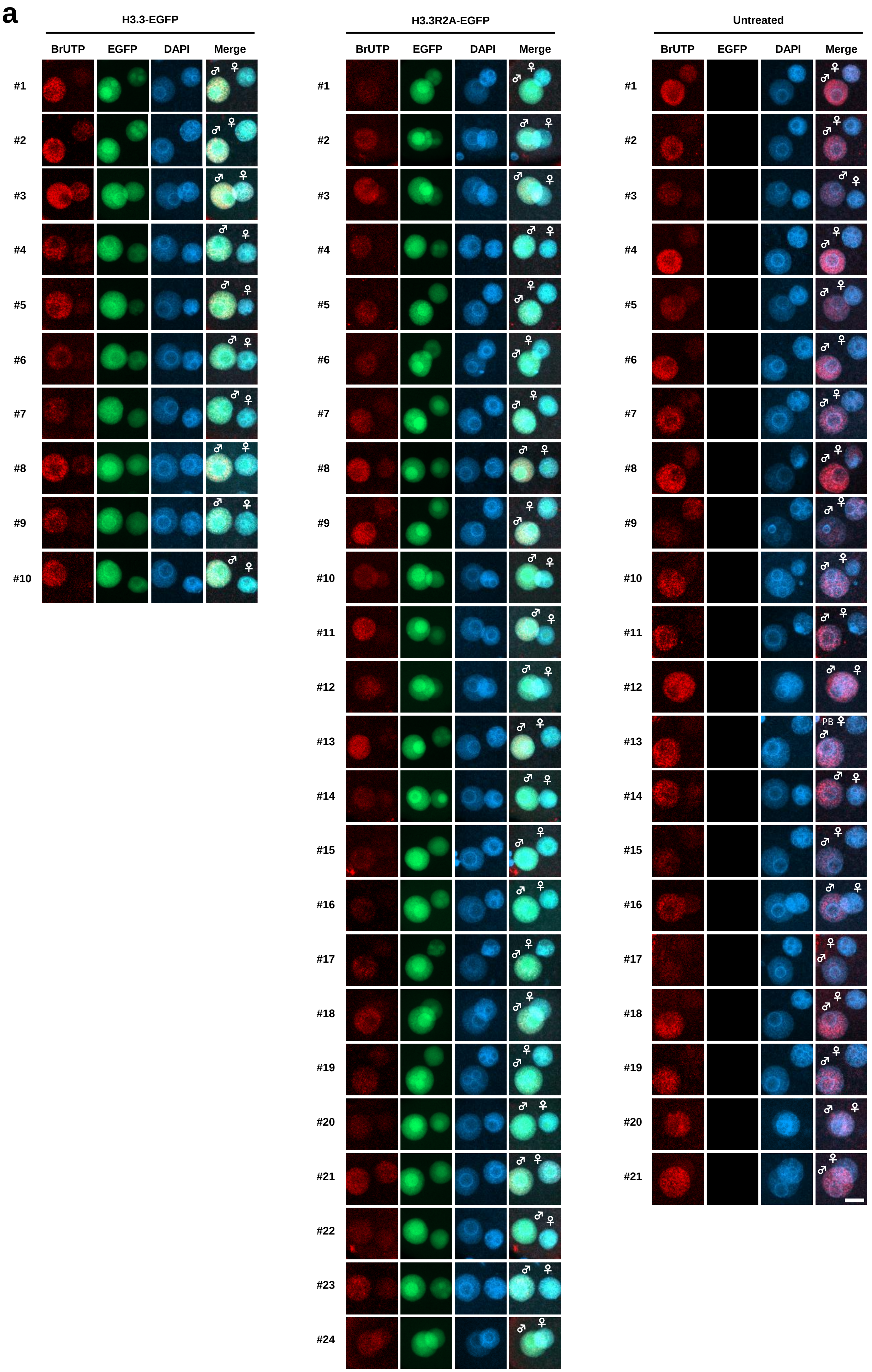
a
H3.3-EGFP
H3.3R2A-EGFP
Untreated
BrUTP
EGFP
DAPI
Merge
#1
#2
#3
#4
#5
#6
#7
#8
#9
#10
#11
#12
#13
#14
#15
#16
#17
#18
#19
#20
#21
#22
#23
#24
♂
♀
PB

b

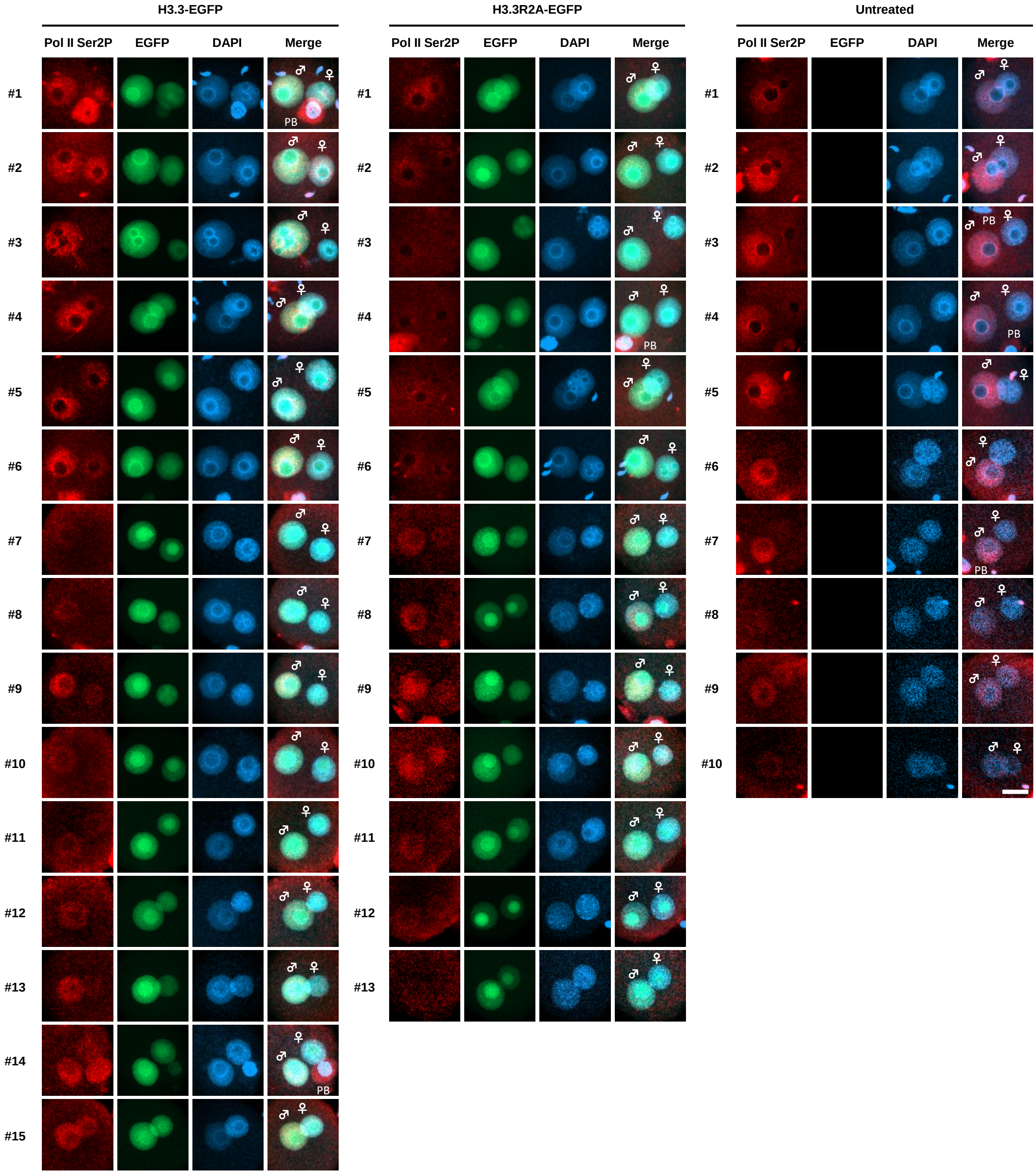
H3.3-EGFP
H3.3R2A-EGFP
Untreated
Pol II Ser2P
EGFP
DAPI
Merge
#1
#2
#3
#4
#5
#6
#7
#8
#9
#10
#11
#12
#13
#14
#15
♂
♀
PB

c

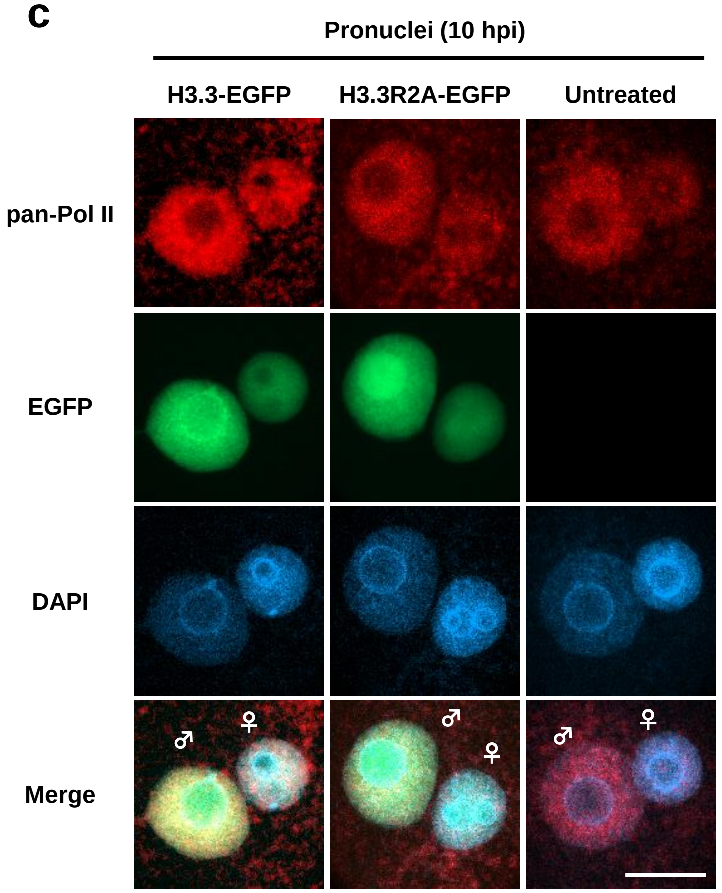
Pronuclei (10 hpi)
H3.3-EGFP
H3.3R2A-EGFP
Untreated
pan-Pol II
EGFP
DAPI
Merge
♂
♀

d

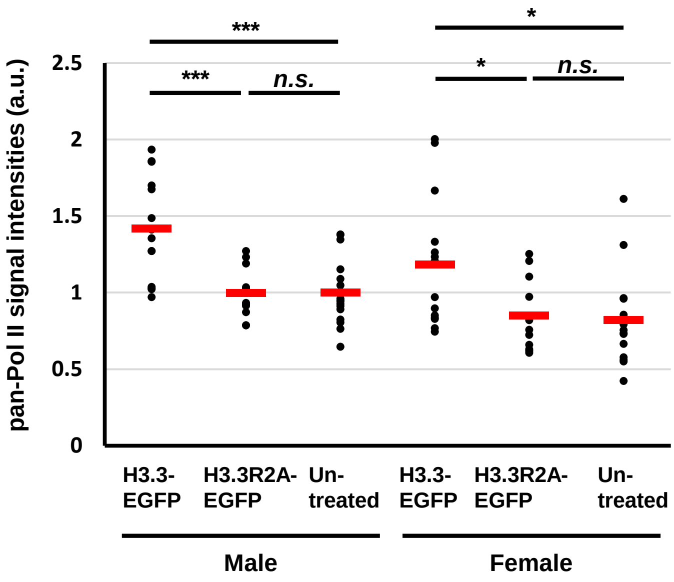
pan-Pol II signal intensities (a.u.)
2.5
2
1.5
1
0.5
0
***
***
n.s.
*
*
n.s.
H3.3-EGFP
H3.3R2A-EGFP
Un-treated
H3.3-EGFP
H3.3R2A-EGFP
Un-treated
Male
Female

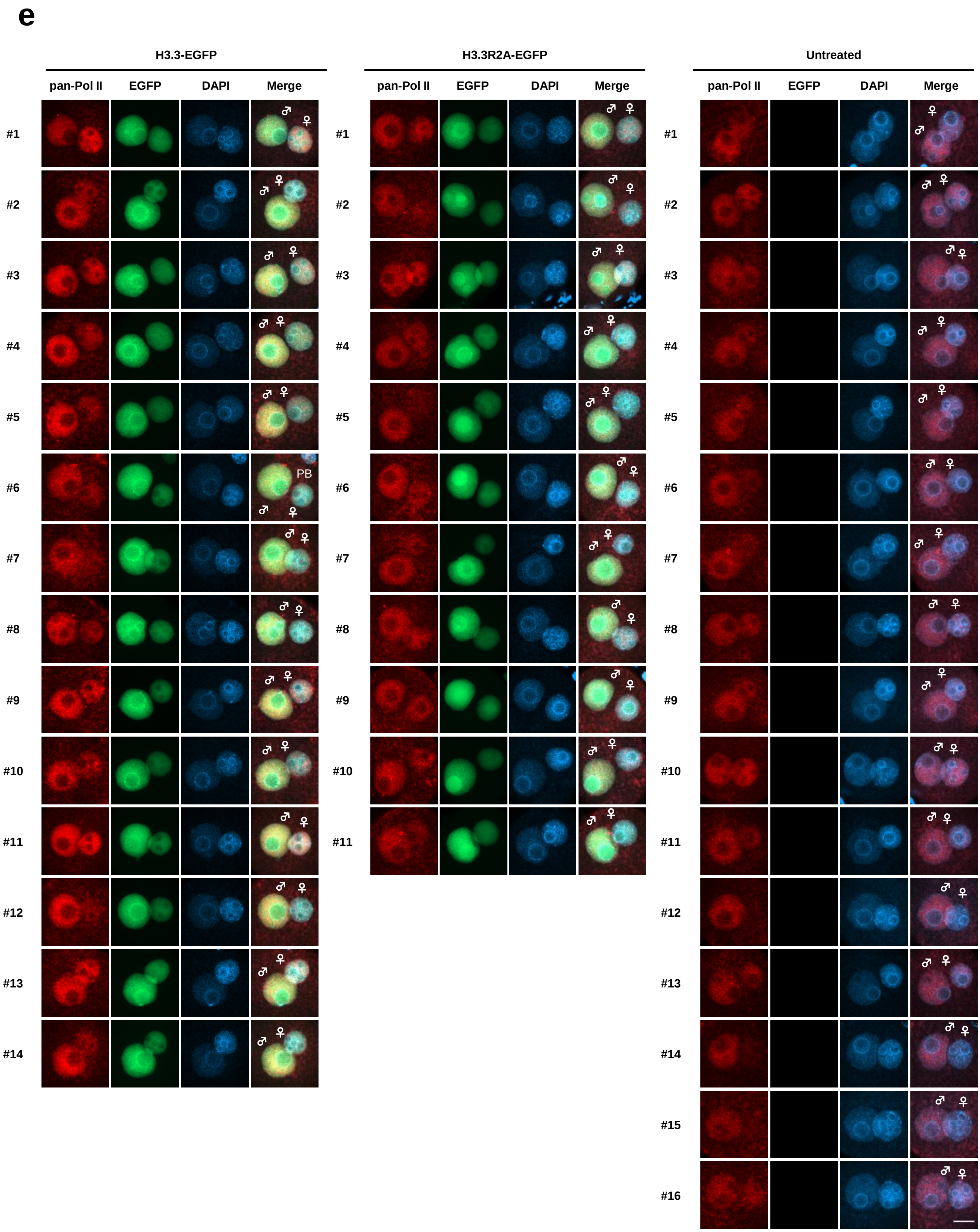
e
H3.3-EGFP
H3.3R2A-EGFP
Untreated
pan-Pol II
EGFP
DAPI
Merge
#1
#2
#3
#4
#5
#6
#7
#8
#9
#10
#11
#12
#13
#14
#15
#16
PB
♂
♀

Fig. S7. Images of BrUTP, Pol II Ser2P and pan-Pol II staining in H3.3-EGFP-expressed, H3.3R2A-EGFP-expressed, and untreated zygotes at 10 hpi. (a) Images of BrUTP (red) stained zygotes for Fig. 3b. (b) Images of Pol II Ser2P (red) stained zygotes for Fig. 3d. (c) Representative immunofluorescent images of pan-Pol II (red) and DAPI (blue) in H3.3-EGFP-expressed, H3.3R2A-EGFP-expressed, and untreated zygotes. EGFP (green) shows successful injection. (d) Quantification of pan-Pol II signal intensities in the pronuclei of H3.3-EGFP-expressed, H3.3R2A-EGFP-expressed, and untreated zygotes. Each dot plot represents a single zygote. Red bars indicate the mean values. The mean value of male pronuclei in untreated zygotes was set as 1. One-way ANOVA and the Tukey–Kramer test were performed to evaluate statistical significance. *Significantly different from the control (*$p < 0.01$, ***$p < 0.001$); *n.s.*: Not significantly different from the control. The number of zygotes was 14 for the H3.3-EGFP group, 11 for the H3.3R2A-EGFP group, and 16 for the untreated group. (e) Images of pan-Pol II (red) stained zygotes for (d). EGFP (green) shows successful injection. DAPI is indicated as DNA. Key: ♂, male pronuclei; ♀, female pronuclei; PB, polar body. Scale bar = 20 μm.

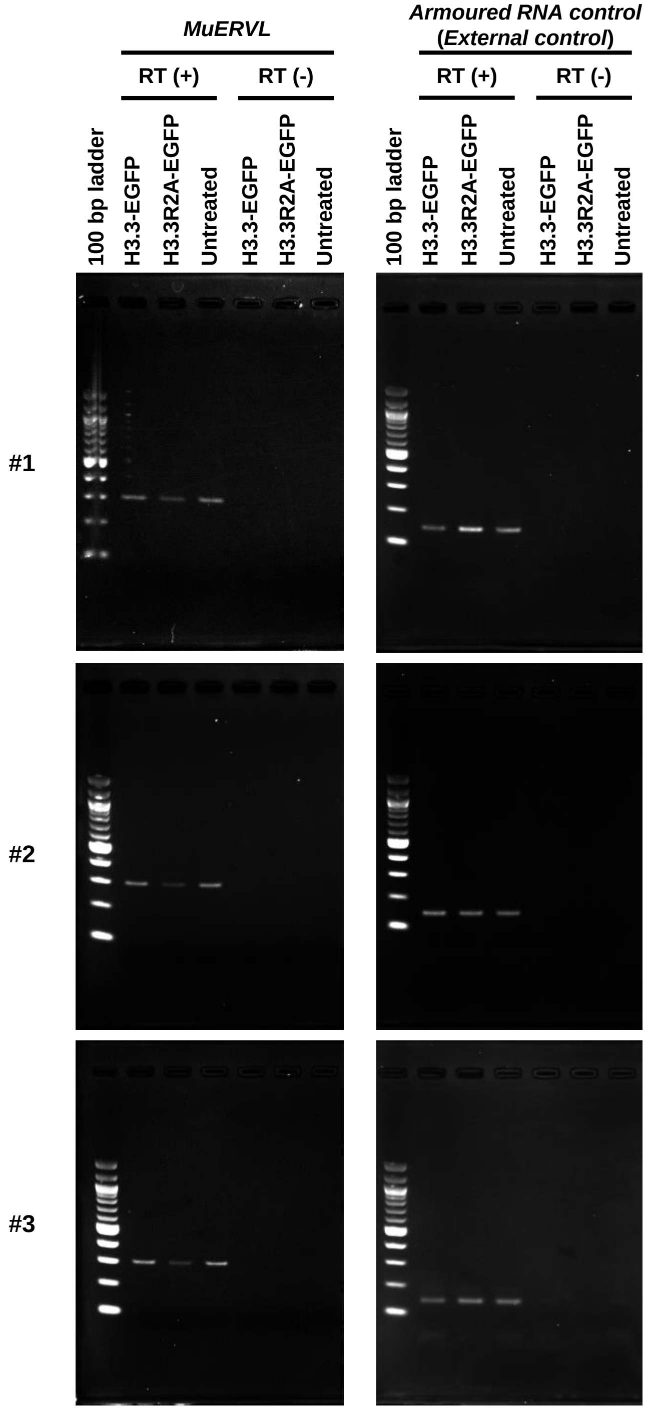

Fig. S8. Images of PCR bands in H3.3-EGFP-expressed, H3.3R2A-EGFP-expressed, and untreated zygotes at 24 hpi. Images of full-length agarose gels for Fig. 4a, b.

a

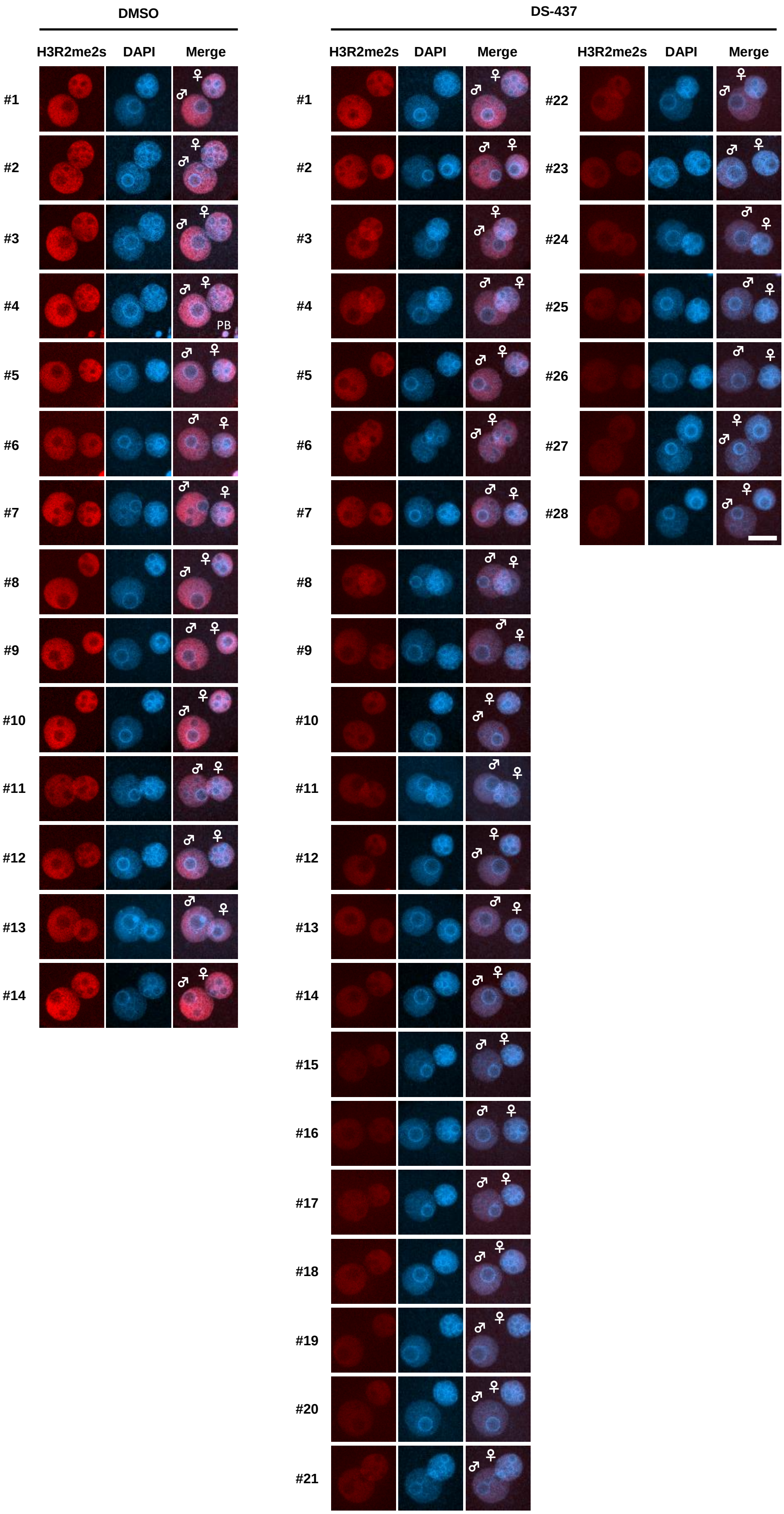
DMSO
DS-437
H3R2me2s
DAPI
Merge
#1
#2
#3
#4
#5
#6
#7
#8
#9
#10
#11
#12
#13
#14
#15
#16
#17
#18
#19
#20
#21
#22
#23
#24
#25
#26
#27
#28
♂
♀
PB

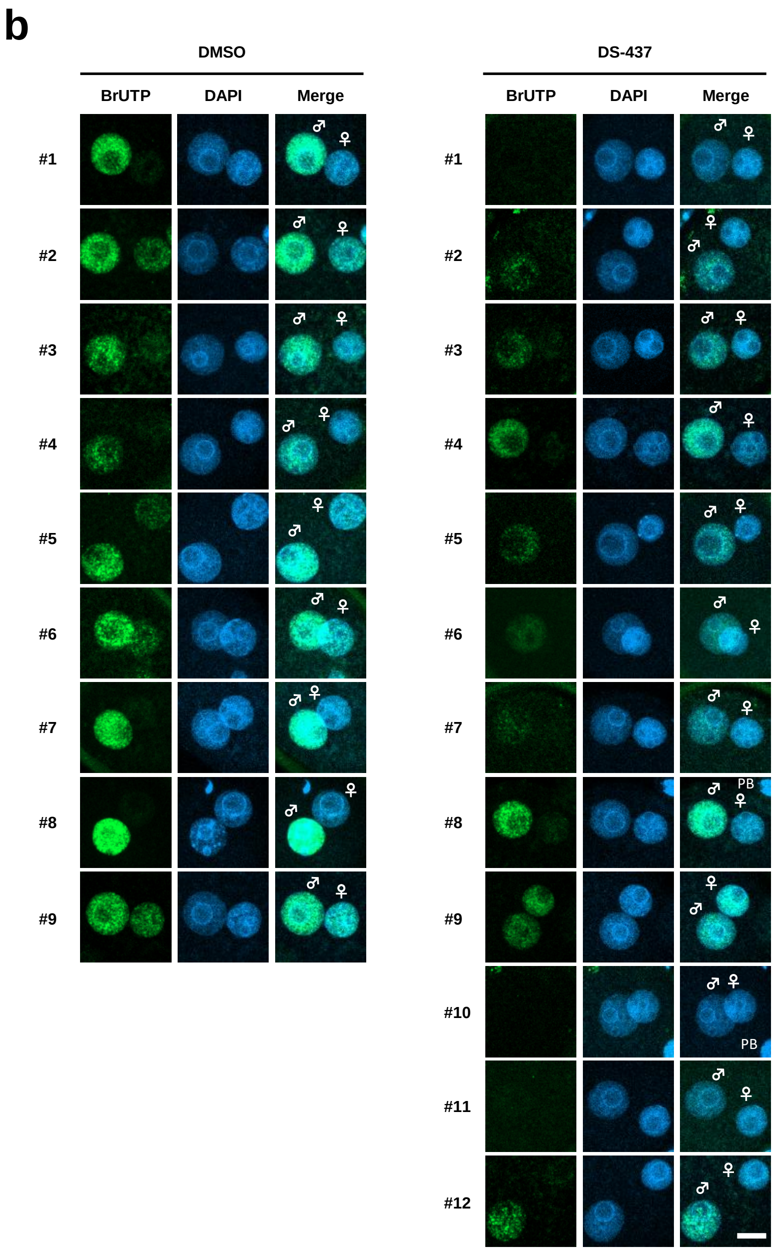
b
DMSO
DS-437
BrUTP
DAPI
Merge
#1
#2
#3
#4
#5
#6
#7
#8
#9
#10
#11
#12
♂
♀
PB

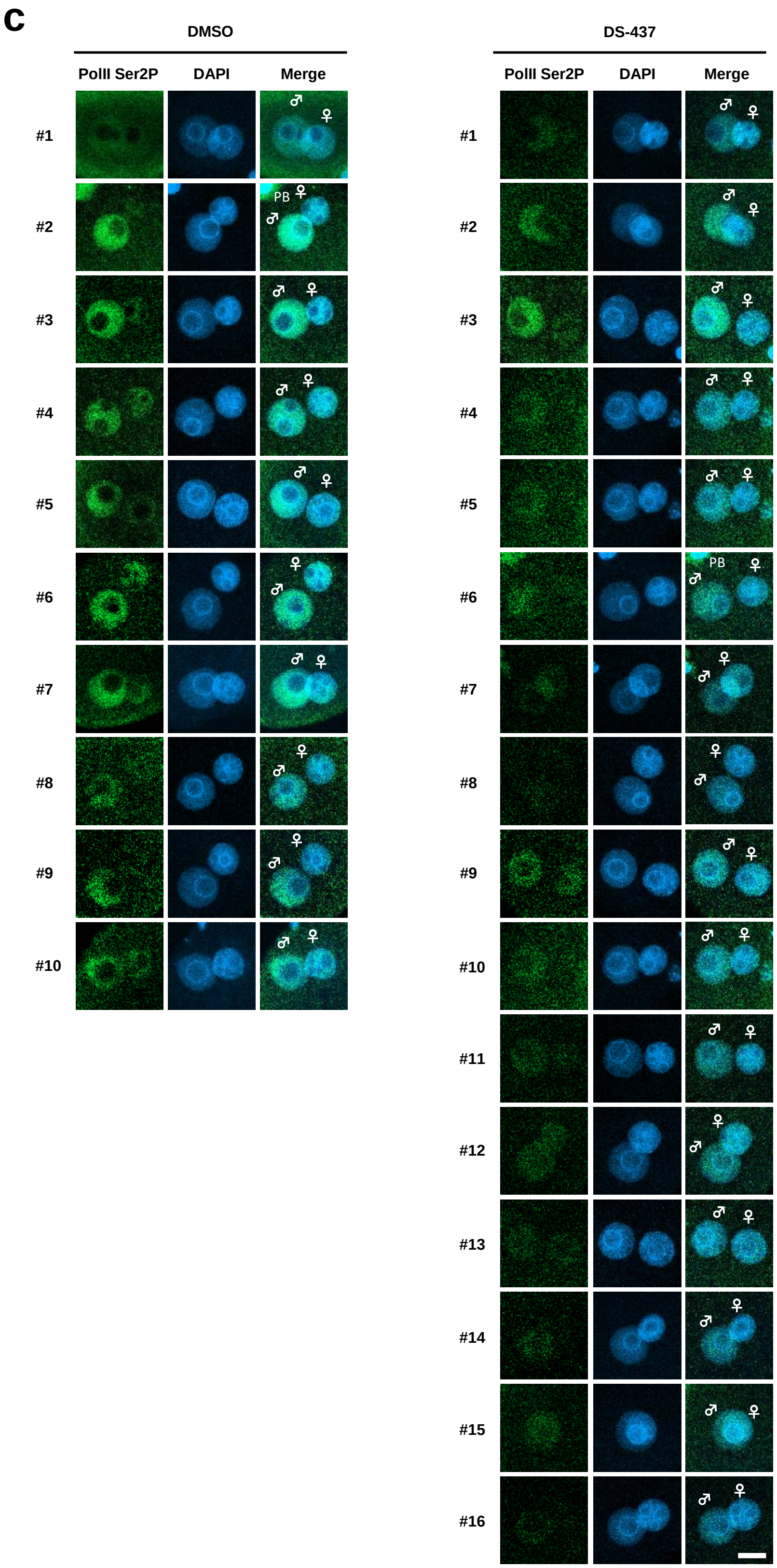

Fig. S9. Images of H3R2me2s, BrUTP, and Pol II Ser2P staining in DMSO- and DS-437-treated zygotes at 10 hpi. (a) Images of H3R2me2s (red)-stained zygotes for Fig. 5c. (b) Images of BrUTP (green)-stained zygotes for Fig. 5e. (c) Images of Pol II Ser2P (green)-stained zygotes for Fig. 5g. DAPI is indicated as DNA. Key: ♂, male pronuclei; ♀, female pronuclei; PB, polar body. Scale bar = 20 μm.

a

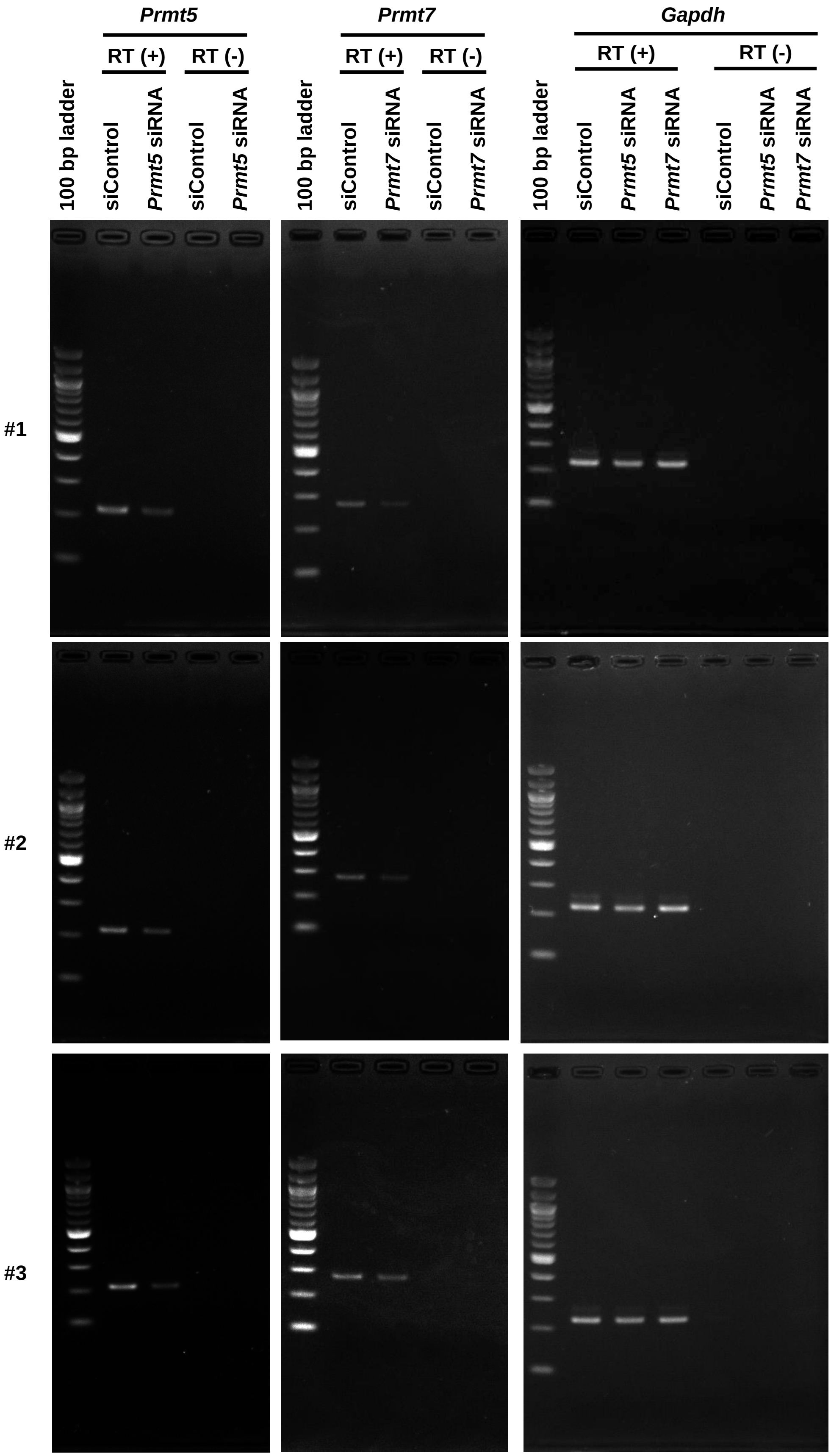
Prmt5
RT (+)
RT (-)
100 bp ladder
siControl
Prmt5 siRNA
siControl
Prmt5 siRNA
Prmt7
RT (+)
RT (-)
100 bp ladder
siControl
Prmt7 siRNA
siControl
Prmt7 siRNA
Gapdh
RT (+)
RT (-)
100 bp ladder
siControl
Prmt5 siRNA
Prmt7 siRNA
siControl
Prmt5 siRNA
Prmt7 siRNA
#1
#2
#3

b

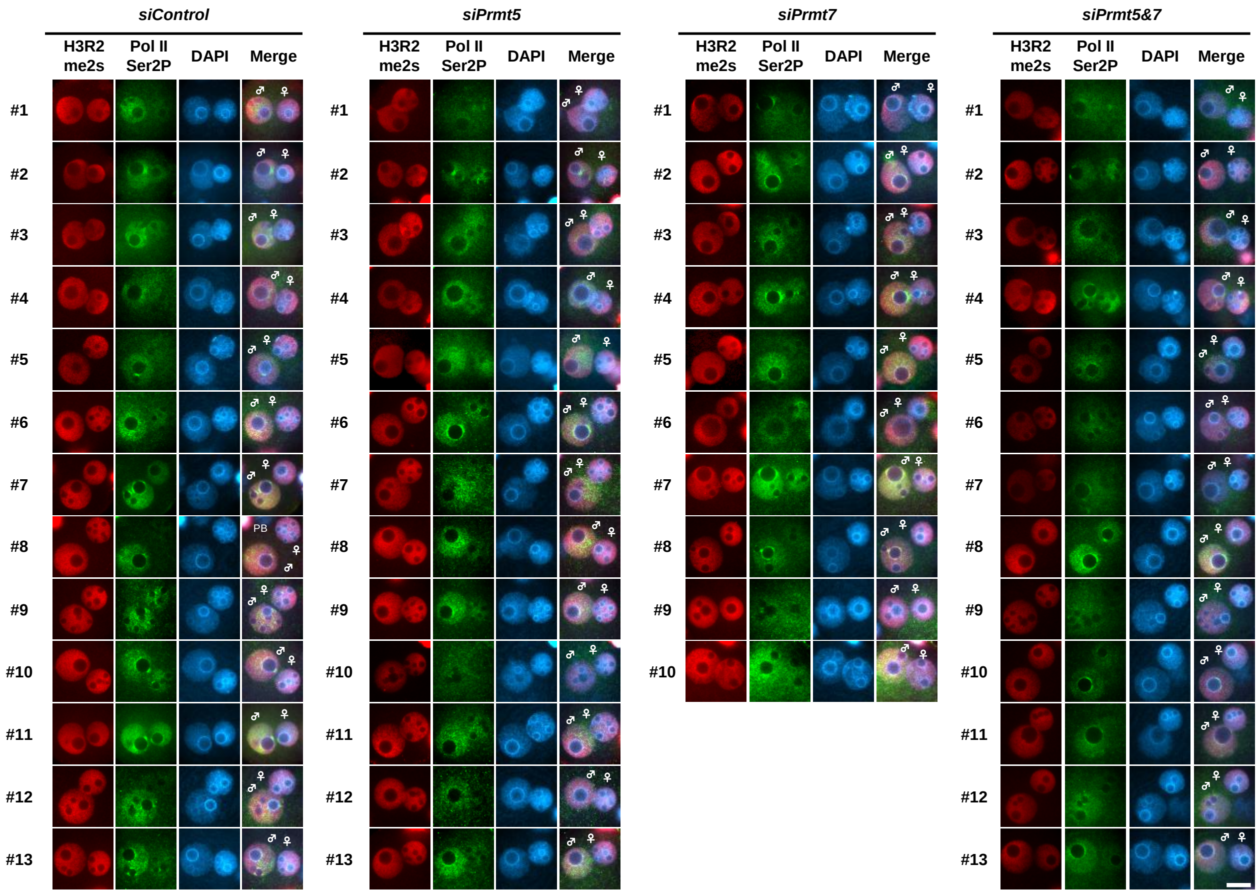

Fig. S10. Images of PCR bands and immunostaining in *Prmt5* and/or *Prmt7* mRNA knockdown oocytes and zygotes at 10 hpi. (a) Images of full-length agarose gels for Fig. 6b. (b) Images of H3R2me2s (red)-stained and Pol II Ser2P (green)-stained zygotes for Fig. 6d, e. DAPI (blue) is indicated as DNA. Key: ♂, male pronuclei; ♀, female pronuclei; PB, polar body. Scale bar = 20 μm.

a

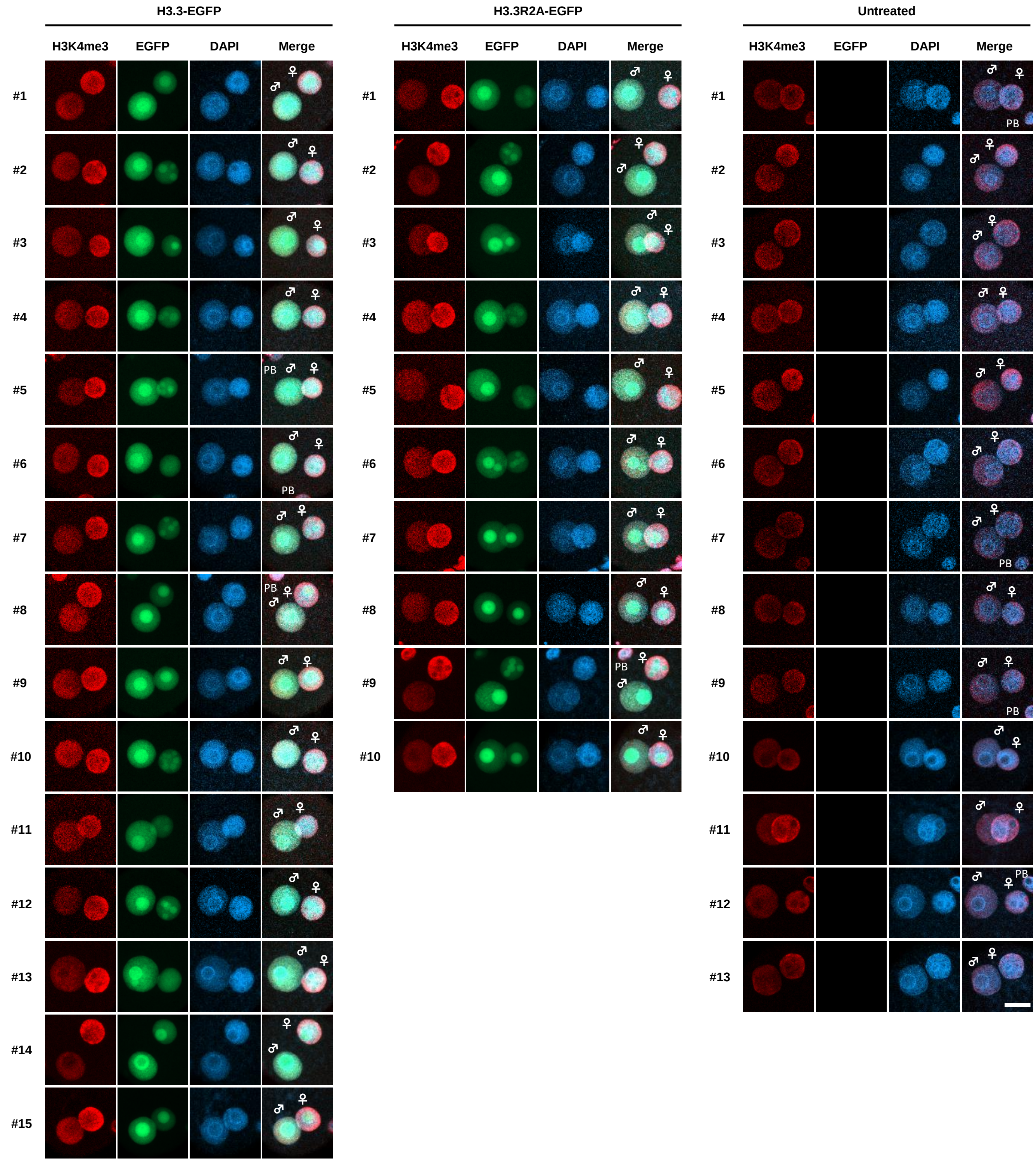
H3.3-EGFP
H3.3R2A-EGFP
Untreated
H3K4me3
EGFP
DAPI
Merge
#1
#2
#3
#4
#5
#6
#7
#8
#9
#10
#11
#12
#13
#14
#15
PB
♂
♀

b

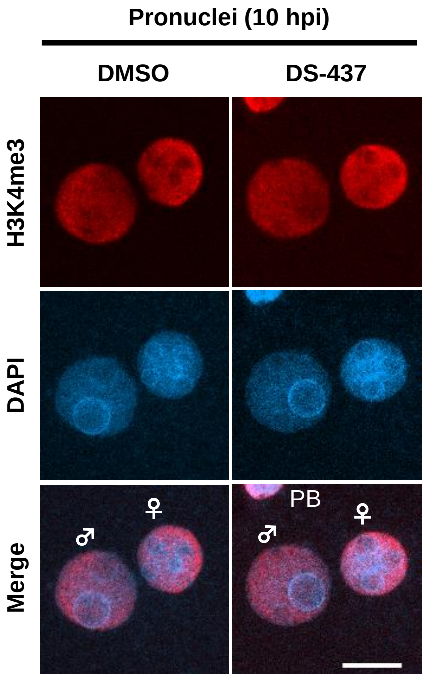
Pronuclei (10 hpi)
DMSO
DS-437
H3K4me3
DAPI
Merge
♂
♀
PB

c

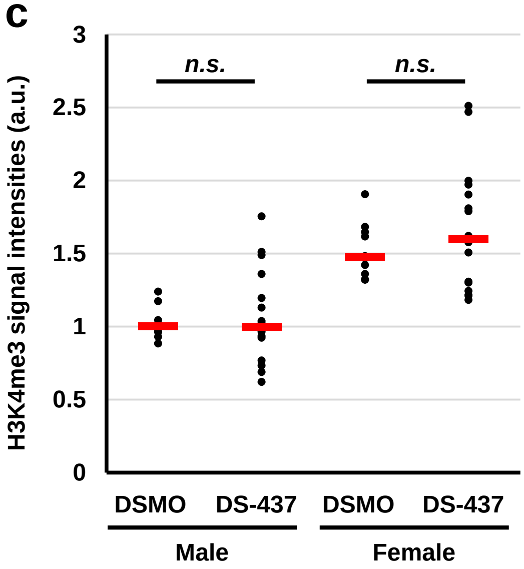
H3K4me3 signal intensities (a.u.)
3
2.5
2
1.5
1
0.5
0
n.s.
n.s.
DSMO
DS-437
DSMO
DS-437
Male
Female

d

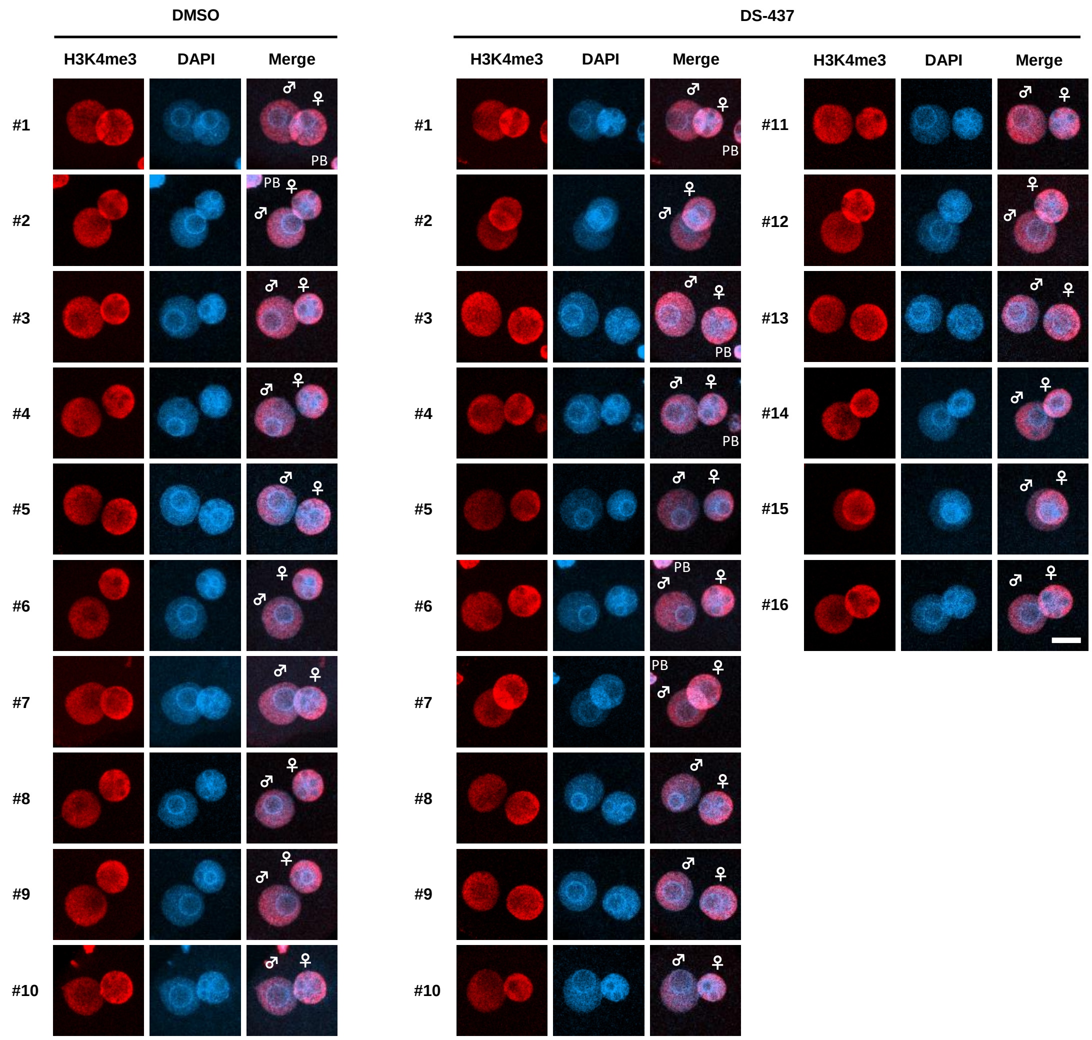

Fig. S11. Images of H3K4me3 staining in H3R2me2s-inhibited zygotes at 10 hpi. (a) Images of H3K4me3 (red) staining in H3.3-EGFP-expressed, H3.3R2A-EGFP-expressed, and untreated zygotes for Fig. 7b. EGFP (green) shows successful injection. (b) Representative immunofluorescent images of H3K4me3 (red) and DAPI (blue) in DMSO- and DS-437-treated zygotes. (c) Quantification of H3K4me3 signal intensities in the pronuclei of DMSO- and DS-437-treated zygotes. Each dot plot represents a single zygote. Red bars indicate the median values. The median value of male pronuclei in DMSO-treated zygotes was set as 1. Mann–Whitney U test was performed to evaluate statistical significance. *n.s.*: Not significantly different from the control. The number of zygotes was 10 for the DMSO group, 16 for the DS-437 group. (d) Images of H3K4me3 (red) staining in DMSO- and DS-437-treated zygotes for (c). DAPI is indicated as DNA. Key: ♂, male pronuclei; ♀, female pronuclei; PB, polar body. Scale bar = 20 μm.

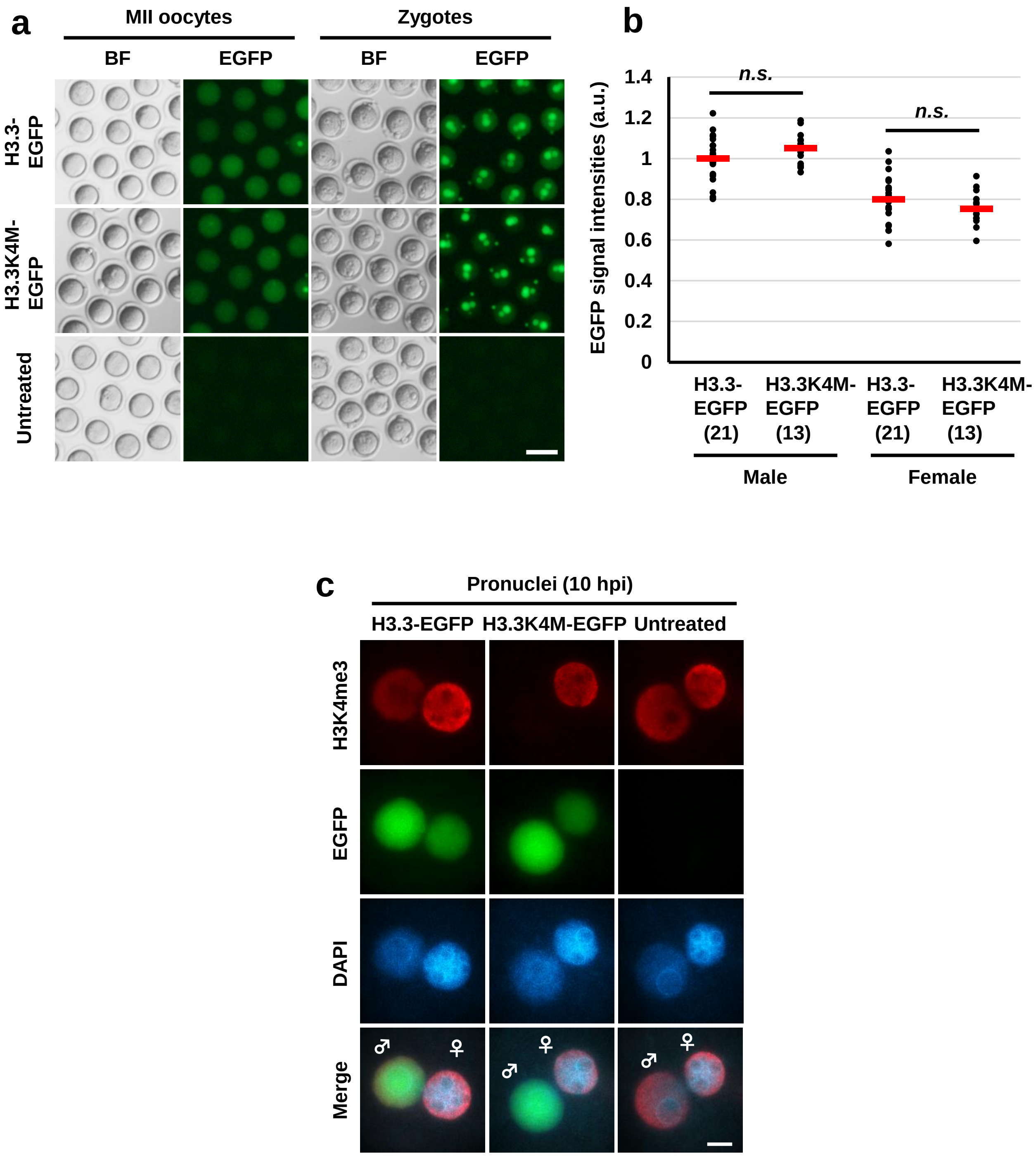

Fig. S12. Inhibition of H3K4me3 by expressing H3.3K4M-EGFP in pronuclei of zygotes. (a) Observation of EGFP-signals in each mRNA-injected zygote. Scale bar = 100 µm. (b) Quantification of EGFP signal intensities in the pronuclei of H3.3-EGFP-expressed, and H3.3K4M-EGFP-expressed zygotes. Each dot plot represents a single zygote. Red bars indicate the mean values. The mean value of male pronuclei in each control zygote was set as 1. Student's t-test was performed to evaluate statistical significance. *n.s.*: Not significantly different from the control. (c) Representative immunofluorescent images of H3K4me3 (red) and DAPI (blue) in H3.3-EGFP-expressed, H3.3K4M-EGFP-expressed, and untreated zygotes. EGFP (green) shows successful injection. Key: ♂, male pronuclei; ♀, female pronuclei. Scale bar = 20 µm.

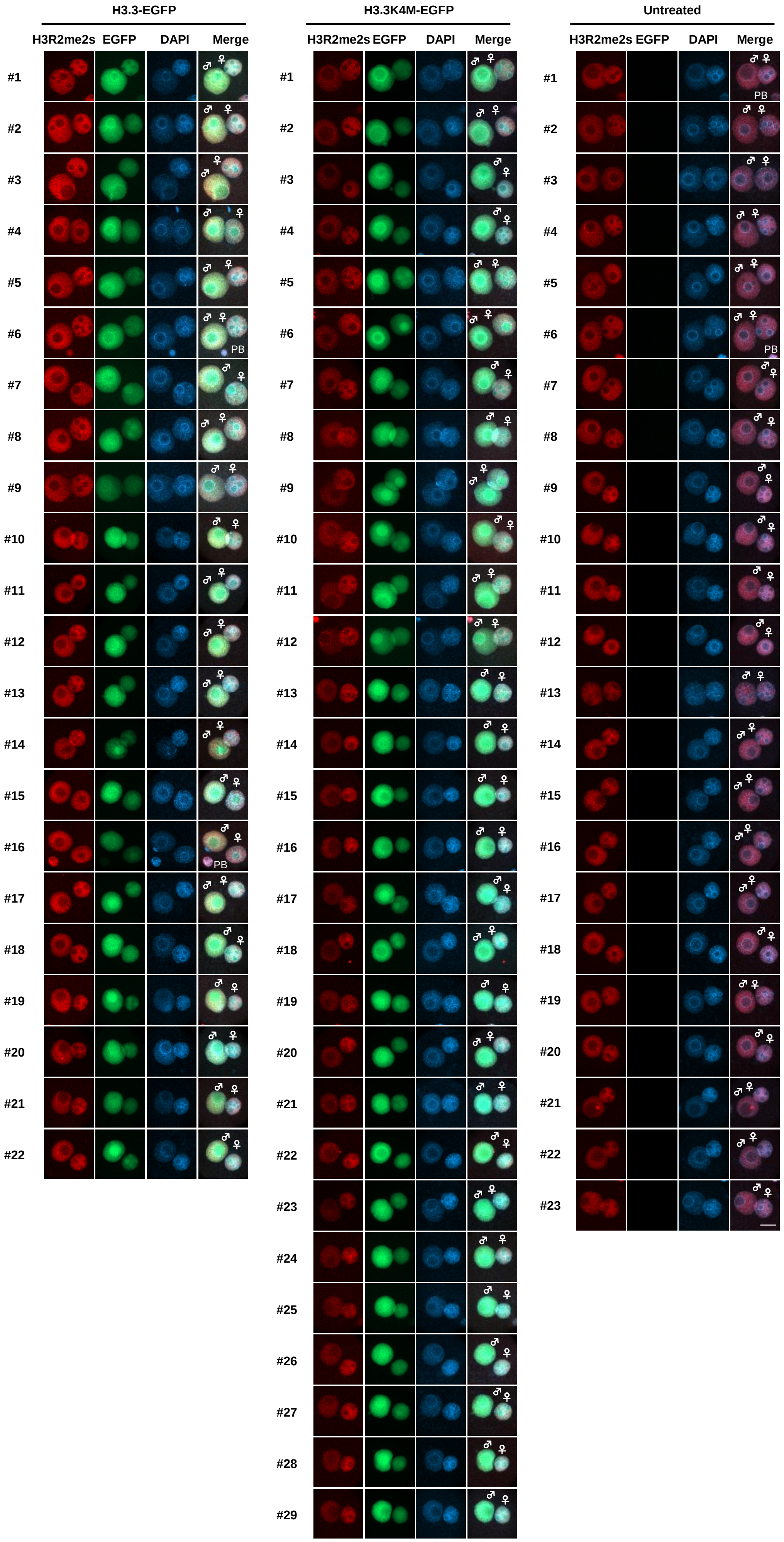

H3.3-EGFP
H3.3K4M-EGFP
Untreated
H3R2me2s
EGFP
DAPI
Merge
#1
#2
#3
#4
#5
#6
#7
#8
#9
#10
#11
#12
#13
#14
#15
#16
#17
#18
#19
#20
#21
#22
#23
#24
#25
#26
#27
#28
#29
♂
♀
PB

Fig. S13. Images of H3R2me2s staining in H3.3-EGFP-expressed, H3.3K4M-EGFP-expressed, and untreated zygotes at 10 hpi. Images of H3R2me2s (red)-stained zygotes for Fig. 7d. EGFP (green) shows successful injection. DAPI is indicated as DNA. Key: ♂, male pronuclei; ♀, female pronuclei; PB, polar body. Scale bar = 20 μm.
